# Management of HIV-Associated Cancers in Sub-Saharan Africa: A Scoping Review of Treatment Outcomes, Challenges, and Strategies for Enhancing Care

**DOI:** 10.3390/idr18050122

**Published:** 2026-10-21

**Authors:** Emmanuel O. Oisakede, Daniel Asogun, Ayobami T. Adegbenro, Godwin-Uzor Treasure Chinazaekpere, Jane Iguma-Asaka, Onoyemeh Hope Adams, Augustina Konadu Larbi-Ampofo, Ehimemen Stacy Aigbokhaevbo

**Affiliations:** 1 Department of Clinical Oncology, Leeds Teaching Hospitals NHS Trust, Leeds LS9 7TF, UK; 2 Department of Health Research, University of Leeds, Leeds LS2 9JT, UK; 3 Department of Genomic Medicine, Barts and The London School of Medicine and Dentistry, Queen Mary University of London, London E1 2AT, UK; 4 Faculty of Health Sciences, Bahçeşehir Cyprus University, Lefkosa-Guzelyurt, Alaykoy, Mersin-10, Turkey; 5 Department of Trauma and Orthopaedics, Chesterfield Royal Hospital, Chesterfield S44 5BL, UK; daniel.asogun@nhs.net; 6 Department of Chemistry and Biochemistry, University of Arizona, Tucson, AZ 85721, USA; atadegbenro@arizona.edu; 7 The Benjamin S. Carson (Snr.) College of Health and Medical Sciences, Babcock University, Ilishan-Remo P.M.B. 4003, Ogun State, Nigeria; godwinuzortreasure@gmail.com; 8 College of Medicine, Ambrose Alli University, Ekpoma P.M.B. 14, Edo State, Nigeria; janenoma04@gmail.com (J.I.-A.); onyadams1@gmail.com (O.H.A.); 9 Acute Medicine, Epsom and St Helier University Hospitals NHS Trust, London SM5 1AA, UK; a.larbi-ampofo@nhs.net; 10 School of Medicine, College of Medical Science, University of Benin, Benin City P.M.B. 1154, Edo State, Nigeria; stacy.aigbokhaevbo@med.uniben.edu

**Keywords:** HIV, cancer, sub-Saharan Africa, treatment outcomes, scoping review

## Abstract

Background: People living with HIV in sub-Saharan Africa face a growing burden of cancer, yet evidence on treatment outcomes and barriers to care is dispersed across cancer types and settings. This review examined treatment approaches, outcomes, challenges, and strategies for improving care. Methods: We conducted a scoping review of primary studies identified through searches of MEDLINE via PubMed, CINAHL, EBSCO, Google Scholar, and African Journals Online, with ClinicalTrials.gov searched for relevant interventional studies. Data on cancer treatment, outcomes, HIV care, barriers, and service strategies were charted and synthesised descriptively. Methodological quality was assessed using the Critical Appraisal Skills Programme (CASP) tools to inform interpretation. Results: Thus, 51 reports from ten countries were included: 13 on Kaposi sarcoma, 13 on cervical cancer, 11 on lymphoma, 8 on breast cancer, and 6 on other cancers or mixed-cancer populations. Selected treatment cohorts reported tumour responses and survival after chemotherapy, radiotherapy, surgery, or combined treatment. Across the care pathway, however, delayed diagnosis, limited treatment capacity, travel and financial costs, toxicity, interrupted treatment, and loss to follow-up constrained outcomes. Associations between HIV status and survival varied by cancer and setting. HIV-specific outcomes were reported inconsistently, and pooled outcomes from studies combining participants with and without HIV could not be attributed to people with HIV alone. Care coordination and patient support were frequently proposed, but few strategies were evaluated comparatively. Conclusions: Improving outcomes requires timely access to complete cancer treatment, coordinated HIV and oncology care, and support for patients throughout treatment. Studies beginning at diagnosis and comparative evaluations of service interventions are needed to establish which approaches improve treatment completion and survival. The study protocol was registered prospectively on the Open Science Framework: 10.17605/OSF.IO/8642R.

## 1. Introduction

Sub-Saharan Africa (SSA) remains the region most affected by HIV. More than 60% of people living with HIV worldwide reside in SSA, where expanded access to antiretroviral therapy (ART) has enabled more people to live longer [1]. This progress has altered the pattern of illness experienced by people living with HIV. Although opportunistic infections and other AIDS-related conditions remain important, longer survival has increased the period during which malignant disease may develop, be diagnosed, and require treatment.

Cancer among people living with HIV is heterogeneous. Kaposi sarcoma, selected subtypes of non-Hodgkin lymphoma, and invasive cervical cancer are conventionally classified as AIDS-defining cancers. Their occurrence is closely related to immunosuppression and infection with oncogenic viruses, including Kaposi sarcoma-associated herpesvirus, Epstein–Barr virus, and high-risk human papillomavirus [2]. ART has reduced the incidence of some AIDS-defining cancers, particularly Kaposi sarcoma and non-Hodgkin lymphoma, but has not eliminated them. At the same time, non-AIDS-defining malignancies, including Hodgkin lymphoma, anal cancer, hepatocellular carcinoma, lung cancer and several head and neck cancers, have become increasingly relevant as people with HIV live longer. Their risk reflects varying contributions from aging, oncogenic infections, residual immune dysfunction, chronic inflammation, behavioural exposures and background population risk [3].

The burden is particularly marked in Africa. A global modelling analysis of cancers attributable to HIV estimated that 70.5% of HIV-attributable cancer cases diagnosed in 2022 occurred in Africa; HIV-attributable malignancies accounted for more than 10% of the overall cancer burden in both Eastern and Southern Africa [4]. Cervical cancer illustrates the scale of this interaction. Women living with HIV have approximately six-times the risk of developing cervical cancer compared with women without HIV, with a particularly high HIV-attributable cancer burden in Eastern and Southern Africa [4,5]. Kaposi sarcoma and HIV-associated lymphomas also remain important causes of morbidity and premature mortality despite wider ART availability [2].

Managing cancer in a person living with HIV requires more than applying standard oncology protocols. Treatment decisions may need to account for immune status, HIV viral suppression, ART history, opportunistic-infection risk, organ function and potential interactions between antiretroviral and anticancer medicines. Drug interactions and overlapping toxicities require coordinated reviews of ART, cancer treatment and supportive medication [2,6]. For HIV-associated lymphoma, guidance recommends continuing effective ART during chemotherapy and modifying interacting medicines when necessary [7]. Cancer treatment may, in turn, disrupt ART adherence, HIV monitoring and continuity of care.

These clinical demands are encountered within cancer systems that have major constraints. Across SSA, access to pathology, imaging, surgery, systemic therapy, radiotherapy, supportive care and specialist oncology personnel remains uneven, while treatment costs and long travel distances frequently fall on patients and their families [8]. For people managing both HIV and cancer, separate HIV and oncology services may create additional appointments, transport costs, referral delays and inconsistent communication between clinical teams. A Ugandan study found that difficulties travelling between multiple facilities and conflicts between HIV and cancer appointments impeded access to treatment [9]. More recently, oncology professionals in Malawi, South Africa, Uganda and Zimbabwe identified limited workspace, staffing, workflow disruption and costs to patients and institutions as barriers to integrating HIV services within cancer centres [10].

There is nevertheless evidence that locally conducted research can improve practice. A multinational randomised trial in Brazil, Kenya, Malawi, South Africa, Uganda, and Zimbabwe demonstrated better progression-free survival with paclitaxel plus ART than with oral etoposide or bleomycin-vincristine plus ART for advanced AIDS-associated Kaposi sarcoma [11]. However, the treatment evidence remains unevenly distributed across malignancies and countries. An earlier assessment of cancer trials in 49 SSA countries identified 96 cancer-related trials, of which only 11 specifically focused on people living with HIV [12]. Existing syntheses have commonly concentrated on individual cancers, clinical trials or broad epidemiological overviews rather than examining treatment, outcomes, delivery barriers and service improvement strategies together.

A scoping review was, therefore, considered appropriate because the evidence spans clinical trials, observational cohorts, qualitative research, programme evaluations and surveys, with considerable variation in cancers, treatments, settings and reported outcomes. In this review, the term HIV-associated cancer was used pragmatically to refer to any invasive malignancy diagnosed in a person living with HIV. It was not intended to imply that HIV caused every included tumour.

This review aimed to map and synthesise evidence on the management of HIV-associated cancers in SSA. Its specific objectives were as follows: (1) describe cancer treatment approaches and the concurrent management of HIV; (2) summarise reported cancer, treatment delivery and HIV-related outcomes; (3) identify patient-level, clinical, institutional and health-system barriers to care; (4) catalogue strategies proposed, implemented or evaluated to improve care; and (5) identify evidence gaps by cancer type, country, population and stage of the care pathway.

## 2. Methods

### 2.1. Review Design and Reporting

A scoping review was conducted using the framework developed by Arksey and O’Malley, refined by Levac et al., and operationalised according to updated JBI guidance for scoping reviews [13,14,15]. The review was reported in accordance with the Preferred Reporting Items for Systematic Reviews and Meta-Analyses Extension for Scoping Reviews (PRISMA-ScR) [16].

The protocol was registered prospectively on the Open Science Framework before formal screening commenced and updated after the screening process: 10.17605/OSF.IO/8642R (https://doi.org/10.17605/OSF.IO/8642R, accessed on 22 September 2026). Any deviations from the registered protocol were documented and justified in the final report.

### 2.2. Review Question

The review was guided by the following question:

What is known about the treatment approaches, outcomes, care delivery challenges and improvement strategies relevant to managing cancer among people living with HIV in sub-Saharan Africa?

Eligibility criteria were developed using the Population–Concept–Context framework.

### 2.3. Eligibility Criteria

#### 2.3.1. Population

Eligible sources concerned children, adolescents or adults living with HIV who had a confirmed diagnosis of an invasive malignancy. Studies of healthcare professionals, caregivers, programme managers or policymakers were also eligible when their findings explicitly concerned the delivery of cancer care to people living with HIV.

Studies containing participants with and without HIV were eligible when HIV-specific findings were reported separately, HIV status was examined in relation to cancer care or outcomes, or the study explicitly addressed cancer management in a population that included people with HIV. Reports meeting the last criterion were retained as contextual mixed-cohort evidence even when outcomes were pooled; those estimates were not interpreted as HIV-specific outcomes. A diagnosis of an AIDS-defining cancer alone was not treated as proof of HIV infection. Studies limited to premalignant disease, including cervical or anal intraepithelial neoplasia, were excluded unless findings for invasive cancer were reported separately.

#### 2.3.2. Concept

The concept of interest was cancer management across the care pathway, including diagnostic confirmation, staging, surgery, radiotherapy, systemic anticancer therapy, ART co-management, opportunistic-infection prophylaxis, supportive and palliative care, follow-up and survivorship.

Eligible outcomes included overall or progression-free survival, mortality, tumour response, recurrence, treatment initiation, delay, completion, interruption or abandonment, adverse events, treatment-related mortality, quality of life, symptom control and healthcare utilisation. HIV-related outcomes during cancer treatment, including ART continuation, adherence, viral suppression, CD4-cell recovery and opportunistic infections, were also eligible.

Studies examining clinical, financial, geographical, sociocultural, organisational or policy barriers were included. Improvement strategies could include integrated HIV–oncology services, referral redesign, patient navigation, multidisciplinary care, workforce development, decentralisation, task sharing, financing mechanisms, treatment-protocol adaptation, supply-chain interventions, telemedicine and data-system strengthening. Screening- or prevention-only studies were excluded unless they addressed linkage to treatment for patients with confirmed invasive cancer.

#### 2.3.3. Context

Eligible evidence originated from countries classified within SSA under the United Nations M49 geographical classification [17]. Studies involving several geographical regions were included only when SSA findings could be extracted separately. Hospital, ambulatory, community, HIV-programme, oncology-programme and palliative-care settings were eligible.

#### 2.3.4. Types of Evidence

Randomised and non-randomised trials, prospective and retrospective cohorts, case–control studies, cross-sectional studies, qualitative studies, mixed-methods studies and programme evaluations were eligible. Case reports and very small case series were considered only when they provide otherwise unavailable information about a management approach or clinically important treatment complication.

Systematic, scoping and narrative reviews were used to locate primary studies and contextualise the findings but were not included as independent units in the treatment outcome synthesis, thereby reducing duplication of primary evidence.

The initial eligible publication window was 1 January 2015 to 17 August 2026, selected a priori to reflect contemporary ART and cancer management practice. Searches were subsequently updated on 31 August 2026. However, no new eligible studies published between 18 and 31 August 2026 were found. No language restriction was applied during searching. Full texts in English, French and Portuguese were reviewed directly or with translation support; potentially eligible publications in other languages were translated sufficiently to assess eligibility and extract the required data.

### 2.4. Information Sources and Search Strategy

MEDLINE via PubMed, CINAHL, EBSCO, Google scholar, and African Journals Online (AJOL) was searched from 1 January 2015 to 17 August 2026. ClinicalTrials.gov was searched for completed, ongoing, or unpublished interventional studies.

Grey literature searches covered WHO, UNAIDS, the International Agency for Research on Cancer, relevant national ministries of health, national cancer-control programmes and major regional HIV or oncology organisations. Papers obtained from this search were utilised for the Discussion section of this study. Reference lists of included studies and relevant reviews were checked, and forward citation searching was undertaken as well. The database searches were updated on 31 August 2026 before final analysis.

The strategy combined controlled vocabulary and free-text terms for three core concepts: HIV infection, cancer and SSA. The geographical block included “Sub-Saharan Africa,” regional terms and the names and historical variants of all eligible countries. Cancer management outcomes or barrier-related terms were not required because these concepts were often absent from titles and abstracts and could have reduced search sensitivity. The supplied PubMed strategy and source-specific search-flow figures are presented in Supplementary Table S1.

The MEDLINE strategy was developed first and adapted to the indexing and syntax of the other databases. A second reviewer (EOO) assessed the strategy using the principles of the PRESS guideline before the final searches were run [18].

### 2.5. Record Management and Study Selection

**Figure 1:**
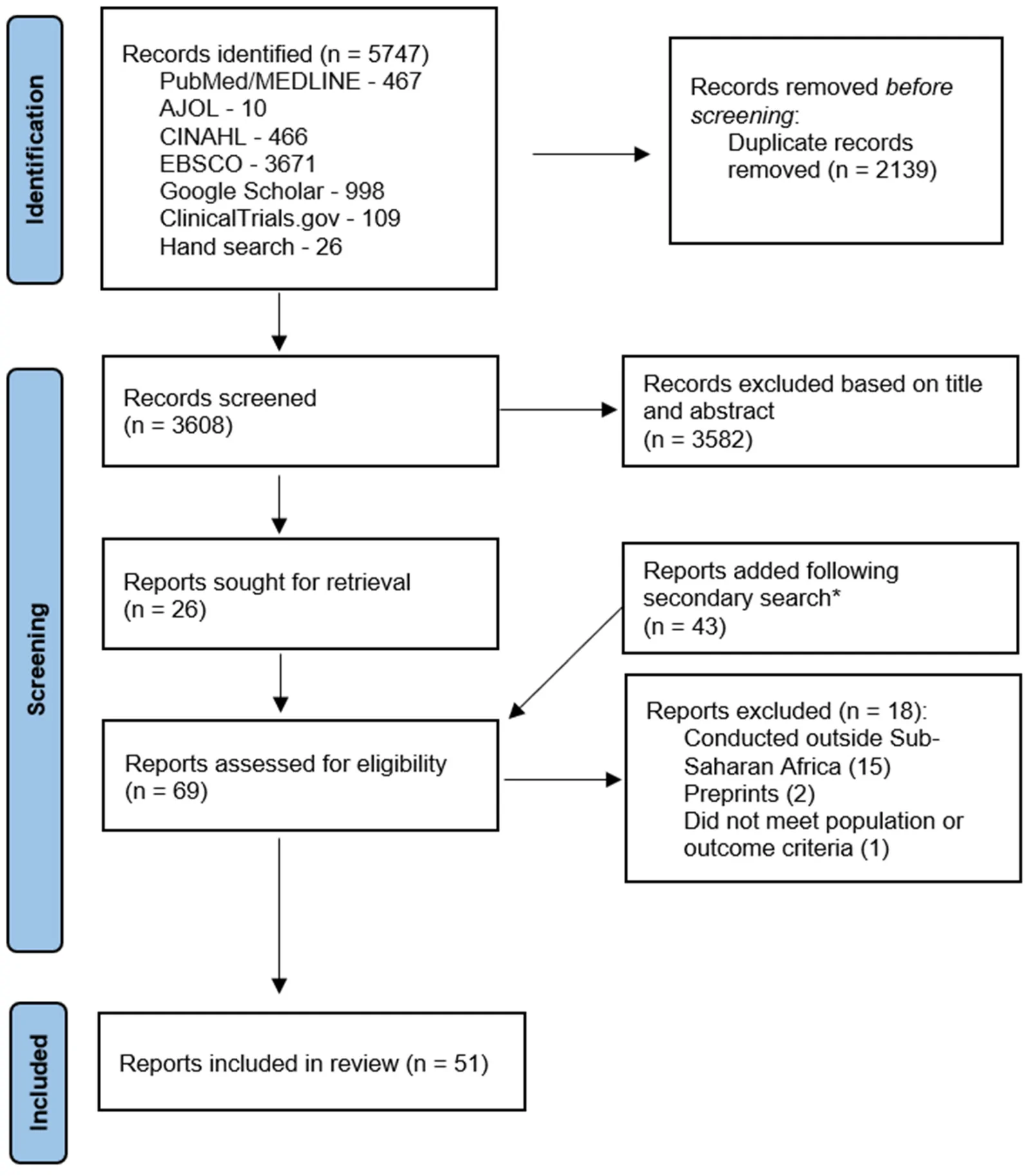
PRISMA-ScR flow diagram of report selection. * The 43 reports obtained through secondary searching were added to the full-text assessment, not automatically included; all 69 reports were subject to the eligibility criteria in Section 2.3.

The reported initial searches identified 5747 records: 467 from PubMed/MEDLINE, 3671 from EBSCO, 466 from CINAHL, 10 from African Journals Online, 998 from Google Scholar, 109 from ClinicalTrials.gov, and 26 through hand searching. After deduplication, 3608 records underwent title/abstract screening and 26 proceeded to full-text retrieval. The 43 additional reports located through subsequent secondary searching entered the same full-text eligibility assessment; they were not included solely because they were cited by another source. The 26 reports from initial screening and 43 additional reports constituted 69 full texts assessed by the reviewers against the same eligibility criteria; 18 were excluded and 51 eligible reports were retained. Reasons for full-text exclusion are presented in Figure 1. Retrieved records were exported to EndNote and deduplicated electronically, followed by manual inspection of author, title, year and digital object identifier fields. Deduplicated records were transferred to Rayyan for screening.

Before formal screening, the review team piloted the eligibility criteria on a sample of records and clarified areas of disagreement. Two reviewers (DA and ATA) then independently assessed titles and abstracts. Full texts of potentially eligible sources were retrieved and independently assessed by the same reviewers. Disagreements at either stage were resolved through discussion and, when consensus could not be reached, adjudication by a third reviewer (EOO). Reasons for full-text exclusion were recorded. Study selection was documented in a PRISMA-ScR flow diagram. See Figure 1.

Multiple publications arising from the same cohort or programme were linked as report families. Complementary endpoints and later follow-up were retained, but confirmed repeat reports were not interpreted as independent populations. Counts describe publications, not a verified number of unique cohorts or participants; no aggregate independent-participant total was calculated. Confirmed and possible links, and their implications for synthesis, are listed in Supplementary Table S2. Shared recruitment sites or overlapping calendar periods were treated as signals of possible overlap, not proof that the same individuals were enrolled.

### 2.6. Data Charting

A standardised data-charting form was developed in Microsoft Excel (v1.0 (M365), UK Enterprise Ltd., Leeds, UK) and piloted on a sample of included studies. The form was refined iteratively without altering the review’s core eligibility criteria or objectives. One reviewer charted the data (EOO), and a second reviewer independently verified all extracted variables against the source publication (DA). Disagreements were resolved by consensus.

The following information was charted:

- Bibliographic details, publication type and funding source;
- Country, study setting, recruitment period and study design;
- Sample size, age group, sex or gender distribution and relevant socioeconomic characteristics;
- HIV diagnostic criteria, ART status and regimen, CD4 count and viral-load information;
- Cancer type, histological confirmation, stage and diagnostic pathway;
- Treatment intent, anticancer regimen, dose or schedule, treatment modification and concurrent HIV management;
- Supportive care, opportunistic-infection prophylaxis and palliative-care provision;
- Outcome definitions, denominators, follow-up periods and effect estimates;
- Treatment delays, completion, abandonment, adverse events and treatment-related mortality;
- HIV-related outcomes during cancer treatment;
- Patient-, provider-, facility- and health-system challenges;
- Strategies proposed, implemented or formally evaluated; and
- Authors’ stated limitations and evidence gaps.

Where information was not reported, it was recorded as “not reported” rather than inferred from contextual information.

### 2.7. Quality of Included Studies

Because this review included heterogeneous study designs and reported clinical outcomes, methodological quality was assessed to support interpretation, although it was not used as an eligibility criterion. Peer-reviewed empirical studies were appraised with the Critical Appraisal Skills Programme (CASP) tool, using the criteria corresponding to each study design. The 2024 design-specific checklists were used [19].

Two reviewers (EOO and ATA) completed the appraisals independently and resolved differences through discussion. Item-level CASP judgments were reported without an overall numerical quality score [19]. Appraisal findings were used to qualify the synthesis and identify recurring methodological weaknesses rather than to exclude evidence.

### 2.8. Data Synthesis and Presentation

The evidence was synthesised into themes because substantial heterogeneity in malignancies, study populations, treatment regimens, outcome definitions and follow-up periods precluded a meaningful pooled effect estimate. Study characteristics were summarised using frequencies, proportions, medians and ranges where appropriate. Counts were reported at the publication/report level unless otherwise stated.

Treatment approaches and outcomes were organised first by AIDS-defining and non-AIDS-defining cancer categories and then by individual malignancy. Each estimate retained its reported population, denominator, endpoint and observation period. Overall survival, progression-free or local disease-free survival, tumour response, and treatment completion were interpreted as distinct outcomes; short-term response was not treated as a surrogate for long-term survival. Estimates with different starting points (diagnosis, treatment initiation or scheduled treatment completion) were not directly compared. Differences in stage, treatment eligibility, censoring and loss to follow-up were considered when interpreting findings. Comparisons were descriptive, with priority given to within-report HIV-stratified or adjusted analyses; survival percentages at different horizons were not ranked or pooled. Effect estimates and measures of uncertainty were presented when available, without recalculating adjusted estimates.

Challenges were coded using an inductive–deductive approach and organised across four levels: patient and household; healthcare professional and clinical team; facility and service delivery; and wider health system and policy. Improvement strategies were linked to their intended barriers and classified as follows: (1) comparatively evaluated interventions; (2) implemented approaches described with observational process or clinical outcomes; or (3) proposed approaches or reported facilitators without intervention-outcome evaluation. Observational outcomes following a programme were not taken to establish the independent effect of its components. These categories distinguish empirical evaluation from recommendations and from evidence of feasibility.

Summary tables present report characteristics and improvement strategies. A country-by-cancer evidence map shows report counts in Table 1; Supplementary Table S3 displays all 48 eligible countries, including those without an included report. Empty/zero cells indicate absence of eligible published evidence in this review, not absence of cancer, treatment or services. The multicountry provider survey is counted once rather than assigned as four independent reports.

## 3. Results

### 3.1. Appraisal of Included Studies

The 51 included reports were appraised using CASP checklists matched to their study designs: 3 randomised trials, 42 longitudinal cohort reports, 2 cross-sectional surveys and 4 qualitative studies (Supplementary Tables S4–S8). Item-level judgments and study-specific rationales are provided in Supplementary Tables S5–S8. No numerical quality score or appraisal threshold was used to exclude studies.

The three randomised reports had different limitations. Busakhala et al. did not describe the allocation sequence/concealment sufficiently to verify it; the flow diagram shows 83 randomised but 70 analysed, and masked response assessment was not documented [20]. Chang et al. used concealed allocation and a masked assessor, but participants/providers were unmasked; 30 were randomised, 1 was subsequently found ineligible, and 25 contributed week-14 outcomes. The small pilot and variable chemotherapy delivery limit precision [21]. Minnaar et al. used HIV-stratified online allocation and masked reporting clinicians/image readers, while participants and treatment staff were unmasked. Of 210 randomised participants, 202 were evaluable at six months; local-control analyses conditioned on survival and available assessment, limiting interpretation of the interim endpoint [22]. Missing or excluded outcomes weaken assigned-group comparisons, and no masking claim was inferred solely from absent reporting.

Several cohorts benefited from prospective recruitment, defined outcomes or adjusted analyses. Across the observational evidence, recruitment at specialist centres, selection after treatment initiation or completion, incomplete follow-up and residual confounding limited causal interpretation. Single-arm treatment series describe outcomes but cannot establish treatment benefit relative to an alternative.

The surveys provided evidence on reported barriers and provider perspectives, rather than observed effects of care strategies. Qualitative studies generally used appropriate interviews and analysis, although reporting of researcher reflexivity varied. Findings from studies combining participants with and without HIV require particular care where outcomes were not reported separately. Reports drawing on potentially shared patient samples were appraised individually but should not be treated as independent cohorts when interpreting the evidence (Supplementary Table S2).

### 3.2. Characteristics of Included Studies

Fifty-one eligible reports published between 2015 and 2026 were included; thirty-two appeared from 2020 onward (Table 1). Fifty reports described work in a single country, and one surveyed oncology personnel across Malawi, South Africa, Uganda and Zimbabwe [10]. Together, the reports covered ten sub-Saharan African countries. Botswana contributed 14 single-country reports, followed by South Africa (10), Malawi (8), Kenya (6), Tanzania (4), Uganda (4), Mozambique (2), Côte d’Ivoire (1) and Nigeria (1). Zimbabwe was represented only in the multicountry survey.

Kaposi sarcoma and cervical cancer each accounted for 13 reports, lymphoma for 11 and breast cancer for 8. The remaining six addressed anal or head and neck cancer, delays across cancer types, engagement in HIV and oncology care, or service integration. Lymphoma reports covered non-Hodgkin lymphoma, Hodgkin lymphoma, Burkitt lymphoma and plasmablastic lymphoma. In the mixed lymphoproliferative cohort reported by Kaimila et al. [23], only the 59 patients with non-Hodgkin lymphoma and 5 with Hodgkin lymphoma contributed to this review; 8 patients with multicentric Castleman disease fell outside its invasive-cancer definition.

Three distinct randomized trial populations were represented: a Kenyan comparison of chemotherapy regimens for Kaposi sarcoma, a Kenyan compression intervention for Kaposi sarcoma-associated leg lymphoedema, and a South African cervical cancer radiotherapy intervention [20,21,22]. Most other clinical reports used prospective or retrospective cohorts, chart reviews, or secondary analyses of established cancer cohorts. Four used qualitative interviews to examine chemotherapy access, treatment adherence or integrated care [24,25,26,27]. Settings ranged from tertiary oncology and radiotherapy centres to HIV treatment networks and a rural programme integrating chemotherapy with antiretroviral therapy [28,29].

Clinical samples varied considerably, from 12 patients in a Malawian plasmablastic lymphoma series to 1573 patients enrolled in a Mozambican Kaposi sarcoma treatment programme [30,31]. Adult populations predominated. Two reports focused on children or young people with Kaposi sarcoma, while some lymphoma cohorts included both children and adults [32,33,34,35]. Several studies enrolled participants both with and without HIV to examine differences in treatment or survival. Where outcomes were reported only for the combined sample, they were retained as mixed-cohort findings and were not attributed specifically to participants living with HIV.

Counts refer to reports, not independent patient populations. Busakhala et al. (2018) and Busakhala et al. (2019) describe response and subsequent survival in the same 70 analysed KS trial participants [20,36]. Grover et al. (2018) and MacDuffie et al. (2021) describe successive follow-up of the same 143-person curative-chemoradiotherapy cohort [37,38]. Other potential links concern Botswana cervical cancer recruitment [39,40,41,42,43,44], the Kamuzu Central Hospital lymphoma programme [23,30,34,45,46,47], Botswana Thabatse breast cancer analyses [48,49], and South African Breast Cancer and HIV Outcomes (SABCHO) analyses [50,51]. Supplementary Table S2 names each report and distinguishes confirmed repeat cohorts from possible or unquantified overlap. Different tumour subsets or endpoints within a programme were not automatically assumed to contain identical participants.

### 3.3. Treatment Approaches and Concurrent HIV Care

#### 3.3.1. AIDS-Defining Malignancies

Across the Kaposi sarcoma (KS) reports, ART was provided alongside cancer treatment. For advanced disease, commonly used chemotherapy included bleomycin–vincristine and doxorubicin–bleomycin–vincristine. In a Kenyan HIV care network, 78% of patients who began chemotherapy received bleomycin–vincristine; in a specialised Mozambican clinic, 78% of those treated received doxorubicin–bleomycin–vincristine. Other reported approaches included paclitaxel, gemcitabine and pegylated liposomal doxorubicin [28,29,31,52]. Access to these regimens varied across programmes, as did the proportion of patients who reached chemotherapy after diagnosis. A Tanzanian series described paclitaxel use in children and young adults with relapsed or refractory KS, or for whom other regimens were unsuitable; 16 of its 17 participants had HIV and received ART [32].

For invasive cervical cancer, treatment centred on external-beam radiotherapy and brachytherapy, frequently with concurrent cisplatin when curative chemoradiation was offered. The studies also described radiotherapy alone, palliative radiotherapy, surgery and a shorter radiotherapy schedule in selected settings. In Botswana, 284 of 519 women (55%) in one cohort initiated curative chemoradiation; HIV status was not associated with its initiation after adjustment, whereas earlier stage and higher haemoglobin were. In Côte d’Ivoire, cancer care was initiated by 54% of women with HIV and 39% of women without HIV [39,40,53,54]. These findings describe access within their respective cohorts and do not establish equivalent access across settings.

For aggressive non-Hodgkin lymphoma (NHL), CHOP was the most frequently described regimen, given with ART to participants with HIV. A Ugandan retrospective cohort also included a small group treated with dose-adjusted EPOCH. Other reports described treatment of Burkitt and plasmablastic lymphoma, with considerable variation in disease presentation and regimens. The Malawian lymphoma programme used pathological confirmation and standardised chemotherapy; its report on HIV outcomes also included patients with multicentric Castleman disease, whose results must be distinguished from those of participants with lymphoma [23,45,46,55,56].

#### 3.3.2. Non-AIDS-Defining and Other Malignancies

Hodgkin lymphoma was generally treated with ABVD, with concurrent ART for participants with HIV [34,47]. Breast cancer care included surgery, systemic chemotherapy and radiotherapy according to clinical indication. South African studies examined treatment completion and chemotherapy dose intensity by HIV status, while a Botswana analysis examined survival among women with HIV receiving different ART regimens. Reports on anal and head and neck cancer described radiotherapy and other cancer-directed treatment in cohorts containing both participants with and without HIV [48,50,57,58,59].

### 3.4. Cancer, Treatment Delivery and HIV-Related Outcomes

Reported outcomes were interpreted within their original denominators, endpoints and time origins. For example, three-year survival in a diagnosis-based cohort and five-year survival among curative-treatment starters answer different questions [37,38,41]; neither percentage establishes superiority of one programme. Response at three or six months, local disease-free survival and overall survival were not treated as interchangeable [22,60]. The following comparisons are descriptive, not pooled or standardised estimates.

#### 3.4.1. Kaposi Sarcoma

Cancer responses were achievable with ART and chemotherapy, but results varied by population and treatment access. In the 70-person analysed sample of a Kenyan randomised trial among participants receiving ART, complete response occurred in 12 of 36 analysed in the gemcitabine arm and 6 of 34 analysed in the bleomycin–vincristine arm; the difference was not statistically significant (*p* = 0.175). Sixty participants were alive at three years, with no demonstrated survival difference between arms. The trial and three-year follow-up concern the same 70 participants [20,36]. In a single-arm Mozambican study of 116 selected patients receiving pegylated liposomal doxorubicin, 92 (80%) achieved complete or partial remission at some point; by 24 months, 23 (20%) had died and 15 (13%) were lost to follow-up [52].

Treatment delivery findings placed these responses in context. In the Kenyan HIV care network, 37% of patients with an indication for KS chemotherapy had initiated it by one month and 56% by one year, with death accounted for as a competing event. Among 1210 patients who began chemotherapy in the earlier Mozambican programme, 43% were lost to follow-up and 8% were known to have died; the latter figure cannot be interpreted as an overall mortality estimate. In a rural Malawian programme combining chemotherapy, ART and community support, estimated 12-month survival was 83% among 114 treated adults, with 77% retained in care. These programme cohorts differed in eligibility, follow-up and available treatment, and the Malawian study did not isolate the effect of integration from other aspects of care [28,29,31].

In Uganda, one-year survival was 64% across a cohort of 200 people with KS, including 166 with HIV and 34 without HIV; HIV-positive KS was associated with a higher hazard of death, and lower CD4 count and higher HIV viral load were adverse markers within the HIV-positive group [61]. A South African retrospective study found lower mortality in a later than an earlier ART period, although period comparisons cannot establish which change in care produced the difference [62]. Smaller Kenyan and paediatric series added response and feasibility data, but outcomes reported for cohorts containing participants without HIV cannot be assigned wholly to those with HIV [32,33,63].

#### 3.4.2. Cervical Cancer

The association between HIV and cervical cancer survival was inconsistent across cohorts. In a Botswana cohort recruited from 2010 to 2015, three-year survival was 35% among women with HIV and 48% among women without HIV; the adjusted hazard ratio for death associated with HIV was 1.95 (95% CI 1.20–3.17). By contrast, a cohort of 143 women who initiated curative chemoradiation, including 96 women with HIV who were all receiving ART, had similar five-year survival by HIV status: 55.1% and 56.8%, respectively. The latter estimate is the later follow-up of the cohort first reported by Grover and colleagues, not a separate patient group [37,38,41].

Subsequent Botswana reports likewise found no significant adjusted survival difference by HIV status among patients receiving curative chemoradiation; they associated outcomes more strongly with stage, haemoglobin or treatment delivered. Their recruitment periods and underlying cohorts may overlap, so their sample sizes and survival estimates were not combined [40,42,43]. In Côte d’Ivoire, two-year survival was 37.9% for the whole mixed HIV-status cohort, and HIV status was not associated with adjusted mortality [53]. In Uganda, however, five-year survival among women who had completed external-beam radiotherapy and brachytherapy was lower for those with HIV under both conventional and shorter radiotherapy schedules. Completion-based eligibility limits can show information about all women starting treatment [54].

Treatment delivery also mattered to early outcomes. In a Tanzanian chemoradiation cohort, completion within 56 days and receipt of at least three cisplatin cycles were associated with a higher likelihood of complete response at three months; HIV status was not significantly associated with that early response [60]. Among women with HIV in Botswana, greater CD4 recovery before cancer diagnosis was associated with lower subsequent mortality, an observational association rather than evidence that a specific ART intervention changed cancer survival [44]. In a South African insurance-claims cohort restricted to 483 women who received cervical cancer treatment within 180 days of diagnosis, 136 had HIV. Compared with women without HIV, women with HIV were more likely to receive radiotherapy (adjusted odds ratio 1.90, 95% CI 1.05–3.45), less likely to undergo surgery (0.53, 95% CI 0.31–0.90), and had higher all-cause mortality (adjusted hazard ratio 1.52, 95% CI 1.06–2.19). Because the cohort included only insured women who received treatment within 180 days, these findings do not describe access or survival among all women diagnosed with cervical cancer [64].

#### 3.4.3. Lymphoma

For NHL, both disease control and treatment toxicity were prominent outcomes. In a prospective Malawian CHOP cohort of 58 patients, 37 had HIV. Twelve-month survival was 45% for the mixed cohort; grade 3–4 neutropenia occurred in 84% of participants with HIV and 31% without HIV, and the report attributed 12 deaths to CHOP toxicity. A separate Malawian report estimated 12-month survival of 48% specifically among 59 participants with HIV and NHL [23,45]. In Uganda, one-year survival was 54.5% among 108 people with HIV treated with CHOP and 80.2% among 12 treated with dose-adjusted EPOCH. The small, non-randomised EPOCH group does not support a causal comparison between regimens [55]. For 26 South African patients with HIV-associated plasmablastic lymphoma treated with CHOP, complete response was 46% and two-year survival was 42% [56].

Results from smaller lymphoma cohorts require the same distinction between stratified and pooled outcomes. A South African Burkitt lymphoma study reported two-year survival of 41% for participants with HIV and 57% for those without HIV, a difference that was not statistically significant. Reported survival for the full cohorts in Malawian Burkitt lymphoma, Hodgkin lymphoma and plasmablastic lymphoma series cannot be presented as HIV-specific survival [30,34,35,46].

Hodgkin lymphoma findings differed across settings. In Botswana, adjusted survival did not differ significantly by HIV status among 78 patients starting treatment (hazard ratio 0.43, 95% CI 0.16–1.16). In a South African prospective cohort, one-year survival was 55% among 31 participants with HIV and 88% among 16 without HIV, with excess early mortality in the HIV-positive group. A Malawian ABVD cohort reported two-year survival of 82% across all 38 participants, 11 of whom had HIV; grade 3–4 neutropenia occurred in 24 of 35 treated participants and delayed treatment in 16. These studies enrolled different populations and do not yield a single estimate of the effect of HIV on Hodgkin lymphoma survival [47,65,66].

#### 3.4.4. Breast and Other Cancers; HIV and Patient-Reported Outcomes

South African breast cancer studies found that comparable delivery of treatment did not necessarily imply comparable survival. In a prospective surgical cohort, 25 of 27 participants with HIV and 106 of 113 without HIV completed chemotherapy, with no significant difference in surgical complications [57]. The non-HIV numerator follows the source paper’s Results and Table 2, which agree with the reported 94% completion rate; its abstract gives an inconsistent numerator. In a separate chemotherapy analysis, median relative dose intensity was 0.89 among 166 women with HIV and 0.87 among 159 without HIV; grade ≥3 anaemia and leukopenia were more frequent in the HIV-positive group [50]. A larger South African cohort associated HIV with poorer overall and disease-free survival and found better survival among women with HIV who were receiving ART at breast cancer diagnosis [51]. These reports should not be summed as independent patient totals where their source populations overlap.

Other measures addressed different parts of survivorship and HIV care. In Cape Town, more than 84% of women who initiated systemic chemotherapy completed it regardless of HIV status, although the HIV-positive comparison group was small [67]. Among survivors of stage I–III breast cancer in Botswana, improvement in physical and mental quality-of-life scores through 24 months after treatment did not differ significantly by HIV status; the analysis excluded women who died before that assessment [49]. A Botswana comparison of 89 women with HIV and oestrogen-receptor-positive breast cancer found similar five-year survival among users of efavirenz-containing and other ART regimens, without establishing equivalence between them [48].

For anal squamous cell carcinoma in Tanzania, complete remission (49%) and two-year survival (86.4%) were reported for a mixed HIV-status cohort of 59 patients; outcomes were not significantly associated with HIV status [58]. In a Botswana head and neck cancer cohort, HIV status was not associated with receipt or delay of definitive radiotherapy, survival or radiotherapy toxicity, but the reported median survival of 36.2 months likewise pertains to the whole cohort [59]. HIV-related outcomes were less consistently measured than cancer outcomes. Among Malawian participants receiving chemotherapy and concurrent ART for lymphoma, CD4 counts rose and HIV RNA declined during follow-up, with patterns differing by diagnosis; these observations do not remove the possibility that patients who remained under follow-up differed from those who did not [23].

### 3.5. Barriers Across the Care Pathway

Patient and household barriers included transport costs, repeated travel, difficulty attending appointments, limited information about chemotherapy, fear, stigma and competing financial demands. In interviews with 100 Ugandan adults with HIV and a new cancer diagnosis, the median interval from first symptoms to cancer treatment was 209 days. Thus, 46% reported difficulty travelling between clinics or hospitals, 23% conflicting HIV and cancer appointments, and 21% prohibitive costs; reporting a barrier was associated with premature discontinuation of cancer treatment [9]. Kenyan interviews with people with HIV-associated KS described financial constraints, travel and scheduling difficulties, and fear or insufficient information about chemotherapy [24]. Breast cancer interviews in Botswana and Tanzania additionally identified intersecting HIV and cancer stigma, nondisclosure and psychosocial strain [25,26].

Clinical and facility barriers appeared at diagnosis, treatment selection and treatment completion. In a Botswana survey of 214 people with cancer, 63.1% reported a diagnostic interval exceeding three months and 50.4% a diagnosis-to-treatment interval exceeding three months. These figures describe the mixed HIV-status survey, not its HIV-positive participants alone [68]. Advanced stage, anaemia and poor performance status constrained curative options in cervical cancer cohorts; myelosuppression and treatment delays complicated lymphoma and breast cancer chemotherapy [39,47,50]. These barriers occurred even where ART was available.

Institutional and health-system barriers concerned the separation of HIV and oncology services, staffing, workspace, treatment availability and the cost of repeated care. In a survey of 195 cancer-care staff across Malawi, South Africa, Uganda and Zimbabwe, respondents identified space, workflow, staffing and costs as obstacles to providing HIV care within cancer centres [10]. Nigerian providers described limited palliative-care training and resources, distance and fragmented referral pathways for women with HIV and cancer. These provider studies documented perceived barriers and opportunities; they did not measure the effect of a new integrated service on patient outcomes [27].

### 3.6. Strategies to Enhance Care and Extent of Evaluation

Table 2 separates comparative intervention evidence from outcomes observed after implementation and from proposals without evaluated clinical outcomes. The three randomised reports tested gemcitabine, compression bandaging and modulated electro-hyperthermia, respectively; their small samples, incomplete outcomes or mixed-HIV subgroup limitations preclude broad claims of effectiveness for people with HIV [20,21,22]. The integrated KS programme, introduction of pegylated liposomal doxorubicin and standardised lymphoma services documented treatment delivery and patient outcomes but had no contemporaneous service-model comparator and cannot isolate the effects of integration, community support or monitoring [29,45,52]. Coordination, navigation, transport/financial support, peer support and palliative-care training were proposed or described as facilitators by patients/providers, rather than demonstrated to improve survival or treatment completion in these reports [9,10,24,25,26,27].

### 3.7. Evidence Gaps Identified by the Review

The evidence was concentrated in KS, cervical cancer, lymphoma and breast cancer, with fewer reports on other malignancies, children, palliative care and care outside established referral programmes. Outcome definitions and follow-up periods differed substantially. Several studies enrolled both people with and without HIV but reported key outcomes only for the combined cohort; others selected patients who initiated, completed or survived treatment. Repeated analyses of the same or potentially overlapping cohorts further limited the number of independent estimates. Comparative evaluations of service-delivery strategies were scarce, while ART continuity, cancer-specific mortality, long-term toxicity, patient-reported outcomes and the effects of proposed integration models were inconsistently measured. These gaps limit comparisons across cancers and countries and prevent a pooled estimate of treatment outcomes specifically for people with HIV. The country-by-cancer distribution is shown in Table 3 and Supplementary Table S3. Thus, 38 of the 48 eligible countries had no included report; Zimbabwe was represented only through the multicountry provider survey, not a country-specific clinical-outcome cohort.

## 4. Discussion

This scoping review of 51 eligible reports shows that outcomes for people living with HIV and cancer in sub-Saharan Africa depend on more than the choice of anticancer regimen. Across cancer types, delays before treatment, incomplete access to recommended treatment, toxicity, and loss to follow-up shaped the outcomes that studies could observe. These findings matter at a population level: an external global analysis estimated that 57,300 of 81,300 cancers attributable to HIV worldwide in 2022 occurred in Africa [4]. That estimate describes disease burden, however, and should not be treated as an estimate of the treatment outcomes measured in this review.

### 4.1. Interpreting Treatment Outcomes Across the Care Pathway

The denominator is central to interpreting the apparent success of treatment. Several included studies followed patients who reached a specialist centre or received a potentially curative regimen. Their survival estimates describe outcomes after access to that care. In contrast, Freeman et al. [28] reported that only 37% of patients with an indication for chemotherapy for Kaposi sarcoma had started it within one month. An external population-based study of 632 women with cervical cancer across sub-Saharan Africa found that 15.8% received cancer-directed treatment with curative potential and 5.2% received guideline-adherent treatment [69]. Its population was not stratified by HIV status, so those percentages cannot be assigned to women with HIV. The comparison nevertheless shows why favourable outcomes among selected treated patients cannot establish that effective treatment is reaching everyone who needs it.

For Kaposi sarcoma, the included reports demonstrate that systemic therapy can produce responses in African settings, while exposing major differences in treatment access and follow-up. The single-arm pegylated liposomal doxorubicin study by Coldiron et al. [52] supports its feasibility; it cannot establish comparative effectiveness against locally available regimens. Loss to follow-up in other cohorts also prevents observed deaths from being interpreted as complete mortality estimates. Current WHO guidance suggests paclitaxel or pegylated liposomal doxorubicin when pharmacological treatment is indicated [70]. The gap between these options and the regimens commonly documented in the included studies raises practical questions about procurement, infusion capacity, toxicity monitoring, and affordability. Local comparisons should measure treatment initiation and completion alongside tumour response.

For cervical cancer, the differing associations between HIV status and survival warrant careful interpretation. Dryden-Peterson et al. [41] observed poorer survival among women with HIV, whereas the selected cohorts receiving curative chemoradiotherapy reported by Grover et al. [37] and MacDuffie et al. [38] found similar survival by HIV status. These results need not be contradictory: they condition on different points in the care pathway and may differ in stage, access to radiation and brachytherapy, treatment completion, and HIV control. They do not establish that HIV has no effect once treatment begins, or that HIV alone explains the survival gap observed in broader cohorts. External WHO guidance places diagnosis, staging, treatment, palliation, and survivorship within the same cervical cancer care pathway [71]. Expanding screening without the capacity to treat invasive disease would leave a critical part of that pathway unresolved.

Using the WHO cervical cancer pathway as an interpretive benchmark, the Botswana cohort initiating curative chemoradiation with brachytherapy demonstrates delivery of definitive treatment and longer-term follow-up, but only among women who reached that treatment [37,38,71]. Earlier pathway losses remain substantial. For example, 284 of 519 women in another Botswana report initiated curative treatment, while access to any cancer care was 42.2% in the Côte d’Ivoire cohort [39,53]. The reviewed patient and provider accounts identify referral, travel and appointment barriers, and the Nigerian palliative-care study identifies training and resource constraints [9,27]. Thus, treatment delivery at selected centres is a documented strength, while diagnosis-to-treatment access and palliative capacity remain gaps. These observations do not constitute a formal WHO-compliance assessment of any programme, nor do treatment-centre survival estimates measure completion of the entire pathway.

The lymphoma studies show both the potential value and the difficulty of delivering intensive treatment. The marked neutropenia and treatment-related deaths reported with CHOP in Malawi make supportive care central to assessing a regimen’s effectiveness [45]. The higher one-year survival observed with dose-adjusted EPOCH than CHOP in the small Ugandan comparison is hypothesis-generating: treatment was not randomly assigned and only 12 patients received EPOCH [55]. Outside this review, a pooled analysis of 1546 participants in prospective HIV-associated non-Hodgkin lymphoma trials associated rituximab use with higher complete response and longer progression-free and overall survival [72]. Trial selection, lymphoma subtype, drug access and supportive-care capacity limit direct transfer of those estimates to the reviewed settings. Subtype-appropriate treatment, therefore, needs to be evaluated together with the diagnostic and supportive services required for safe delivery.

The higher haematological toxicity in the Malawian CHOP cohort and South African breast chemotherapy analysis supports a dedicated safety pathway, not automatic dose attenuation based on HIV status [45,50]. In resource-limited settings, practical priorities include reliable blood-count monitoring, clear instructions to seek urgent care for fever, a rapid infection assessment/treatment pathway, and access or referral arrangements for blood products and serious complications. These are clinical implications of the observed harms; their benefit was not tested as a package in the included reports. ASCO’s 2026 guideline supports primary colony-stimulating-factor prophylaxis when febrile-neutropenia risk is approximately 20% or greater and no equally effective, safe alternative regimen is available; at lower regimen risk, patient factors may justify prophylaxis [73]. Grade 3–4 laboratory neutropenia is not synonymous with febrile neutropenia; the 84% neutropenia figure cannot itself determine that threshold. Cost, procurement and emergency-care capacity must be considered when adapting guidance locally.

For breast cancer, similar chemotherapy dose intensity among women with and without HIV in O’Neil et al. [50] suggests that HIV status alone need not determine treatment intensity. That finding must be read alongside greater haematological toxicity in the HIV group and the survival disadvantage reported in Phakathi et al. [51]. An external meta-analysis also found worse adjusted overall survival among women with HIV in sub-Saharan African breast cancer studies [74]. Because that synthesis may share underlying cohorts with the present review, it is contextual evidence rather than independent replication. Comparable chemotherapy delivery and unequal overall survival can coexist; stage, tumour characteristics, HIV control, treatment beyond chemotherapy, and non-cancer deaths require separate assessment before the difference is attributed to any one cause.

### 4.2. HIV Care, Barriers, and Proposed Strategies

CD4 count, viral load and ART status provide related but non-interchangeable clinical information. In the Ugandan KS cohort, lower CD4 count and higher viral load were adverse prognostic markers within the HIV-positive group [61]. In Botswana cervical cancer, greater prediagnosis CD4 recovery was associated with lower mortality in some categories, but the analysis was observational and used incomplete serial measurements [44]. Better breast cancer survival among women already receiving ART at diagnosis likewise represents an association, not a randomised ART effect [51]. Conversely, the selected curative cervical cohort, in which all women with HIV were receiving ART, reported similar survival by HIV status [37,38]. Together, these findings support considering HIV control alongside stage and treatment access, rather than using HIV serostatus as a sufficient prognostic explanation. An external US cohort associated cancer treatment with CD4 declines and linked declining CD4 counts to mortality [75]; it does not establish that reducing indicated cancer therapy would improve outcomes. For lymphoma, EHA–ESMO guidance recommends effective ART during chemotherapy, interaction review and CD4-informed infection prophylaxis [7]. Inconsistent viral-load, adherence and longitudinal CD4 reporting prevents this review from identifying a universal CD4 threshold or an ART regimen that optimises cancer outcomes.

The barriers identified in this review operate at patient, clinical, facility, and health-system levels. Travel costs, repeated visits, stigma, delayed diagnosis, limited treatment capacity, and interruptions in supply can accumulate for the same patient. An external synthesis of cancer-care delays across sub-Saharan Africa identified similar financial, referral, and service-capacity obstacles beyond HIV-associated cancers [76]. Furthermore, a population-based US study found lower receipt of treatment for several cancers among people with HIV, even in a higher-resource setting [77]. These comparisons support examining how services respond to HIV status as well as patients’ clinical needs; neither comparison proves the cause of a disparity in the included African cohorts.

Coordinating oncology and HIV care is a plausible response to fragmented appointments, drug interaction concerns, and missed referrals, but the included studies provide limited comparative evidence that a particular integration model improves cancer survival. An external systematic review of HIV integration with other services found improvements in several ART care outcomes, without a significant pooled mortality benefit; most evaluated services were outside oncology [78]. The reviewed accounts of navigation, community support, and coordinated care, therefore, justify implementation and evaluation, rather than a claim of proven cancer benefit. The limited evidence for adjunctive interventions in the included studies likewise argues for preserving a distinction between a strategy that was proposed, one that was delivered, and one shown to improve outcomes.

### 4.3. Recommendations for Practice

- Track access from diagnosis through completion. Cancer and HIV services should record the dates of diagnosis, staging, referral, treatment initiation, interruptions, completion, and follow-up. Recording patients who never start treatment is necessary to detect gaps hidden by treatment-centre survival estimates. The authors clearly state that these are service-monitoring recommendations informed by the reported access gaps, not interventions shown to improve survival within this review [9,28,39,68].
- Coordinate cancer treatment with HIV care. Oncology and HIV clinicians should review ART, viral load and CD4 results, where available, potential drug interactions, infection risk, and toxicity monitoring as part of one treatment plan. HIV status should prompt an individual clinical assessment rather than an automatic reduction in anticancer treatment. Continuation of effective ART with regimen-specific interaction review is consistent with HIV-associated lymphoma guidance [6,7].
- Strengthen haematological supportive care. For people receiving myelosuppressive chemotherapy, establish baseline and pre-cycle blood counts, additional checks when clinically indicated, fever education and an urgent referral pathway; ensure timely infection management and access to transfusion support when required. Consider granulocyte colony-stimulating factor (G-CSF) according to regimen- and patient-specific febrile-neutropenia risk, rather than HIV status or an isolated neutropenia percentage alone [45,50,73]. For HIV-associated lymphoma, incorporate CD4-informed opportunistic-infection prophylaxis under the relevant guideline and local protocol [7]. These are guideline-informed practice recommendations, not proven service-intervention effects from this scoping review.
- Prioritise complete delivery of established treatment. The cervical cancer findings support reliable access to staging, radiotherapy and brachytherapy; the lymphoma findings support pathology and supportive care alongside chemotherapy. For Kaposi sarcoma, programmes considering the options in current WHO guidance must also plan for procurement, administration, and monitoring. These priorities draw on observed treatment delivery gaps and external guidance rather than direct comparisons of whole-service models [37,38,39,45,70,71].
- Reduce the burden of reaching care. Coordinated appointments, active referral follow-up, clear treatment information, and assistance with travel or treatment costs address barriers repeatedly identified in the included studies. Services should provide these measures in ways that protect HIV confidentiality. Their effectiveness should be evaluated locally because the supporting reports identify barriers/facilitators rather than causal benefits [9,10,24,25,26,27].
- Offer supportive and palliative care throughout the pathway. Symptom control, psychosocial support, and assessment of financial strain are relevant during active treatment as well as advanced disease. The WHO’s framework for invasive cervical cancer care includes palliation and survivorship alongside diagnosis and treatment. These are pathway-based recommendations, while the Nigerian provider study identifies training and resource gaps rather than an evaluated palliative-care intervention [27,71].

### 4.4. Recommendations for Future Research

- Future studies should enrol patients at cancer diagnosis, including those who do not start treatment, and follow them through referral, treatment, and survival. They should report outcomes separately by HIV status in mixed cohorts and, where feasible, capture tumour stage and subtype, ART exposure, viral suppression, CD4 count, treatment intensity, interruptions, cause of death, and loss to follow-up. This would make the meaning of an HIV-associated difference in outcome much clearer.
- Comparative studies should test specific service interventions, such as coordinated appointments, navigation, referral tracking, or transport support against defined usual care. Outcomes should include treatment initiation and completion, cancer and HIV outcomes, patient costs, quality of life, and resource use. For cancer regimens, adequately sized, subtype-specific comparisons with explicit toxicity and supportive-care measures are needed; small non-randomised treatment groups cannot settle relative effectiveness.
- Research should also reach beyond the tertiary centres, countries, and tumour types that dominate the current evidence. Multicentre population-based studies could better represent rural patients, people who never reach oncology services, and cancers with sparse evidence. Finally, investigators using established cohorts should identify overlapping participants across publications so that subsequent reviews can distinguish new patients from longer follow-up of the same patients.

### 4.5. Strengths and Limitations

The breadth of the 51 included reports allowed this review to connect treatment approaches and outcomes with patients’ experiences and service-delivery barriers. The completed design-specific CASP appraisal provides a structured basis for judging the included evidence, but appraisal cannot repair missing outcomes or remove confounding from observational comparisons.

The principal limitation is heterogeneity in cancers, stage, treatment, setting, outcome definitions, and follow-up. Many reports came from referral centres or included only treated patients; their findings cannot be generalised to everyone diagnosed with cancer. Loss to follow-up may distort survival estimates, while all-cause mortality cannot consistently be separated into cancer-related and other deaths. Some included studies combined participants with and without HIV without reporting distinct outcomes, so their pooled estimates cannot be attributed to people with HIV. The 51 publications are not 51 verified independent cohorts. Confirmed repeat cohorts and possible overlap within larger programmes must not be counted as independent patient samples; exact overlap could not be quantified for every family (Supplementary Table S2). Differences in survival horizons and time origins prevent direct ranking of reported percentages. The geographic concentration of evidence and limited evaluation of proposed service strategies further constrain conclusions about the wider region. Accordingly, this review identifies patterns and priorities for care and research, but it does not establish a pooled treatment effect or the causal effect of HIV status on cancer survival.

## 5. Conclusions

This scoping review of 51 publications shows that outcomes for people living with HIV and cancer in sub-Saharan Africa are shaped by access to diagnosis and treatment, treatment completion, toxicity, and continuity of HIV care. Effective treatment is possible, but outcomes reported among patients who reach specialist services cannot represent everyone diagnosed with cancer. Differences in study populations, treatment availability, follow-up, and reporting also prevent a single estimate of the effect of HIV on cancer survival.

Improving care requires reliable access to established cancer treatment, coordination with ART services, supportive care, and practical help for patients navigating referrals and repeated visits. Proposed service strategies are promising, but few have been evaluated for their effect on cancer outcomes. Future studies should follow patients from diagnosis, report HIV-specific outcomes separately, and test interventions that improve treatment initiation, completion, and survival.

## Figures and Tables

**Table 1:** Distribution and principal characteristics of the 51 included reports.

Evidence Focus	Reports (n)	Single-Country Settings	Principal Designs and Subjects Charted
Kaposi sarcoma	13	Kenya (6); Malawi (2); Mozambique (2); South Africa, Tanzania and Uganda (1 each)	Two randomized trial populations, clinical and programme cohorts, and one qualitative study; anticancer treatment, ART co-management, supportive care, treatment access and outcomes
Lymphoma	11	Malawi (6); South Africa (3); Botswana and Uganda (1 each)	Prospective and retrospective cohorts and subtype series; chemotherapy, concurrent ART, toxicity and survival
Cervical cancer	13	Botswana (8); South Africa (2); Côte d’Ivoire, Tanzania and Uganda (1 each)	One randomized trial population, prospective and retrospective cohorts, and a claims-based analysis; treatment access, radiotherapy or chemoradiotherapy, response and survival
Breast cancer	8	South Africa (4); Botswana (3); Tanzania (1)	Clinical cohorts, nested analyses and two qualitative studies; treatment delivery, toxicity, survival, quality of life and care barriers
Other cancers and cross-cancer care	6	Botswana (2); Nigeria, Tanzania and Uganda (1 each); one multicountry report covering Malawi, South Africa, Uganda and Zimbabwe	Anal and head and neck cancer cohorts; patient and provider studies of delays, continuity of care and service integration

**Table 2:** Strategies to enhance care, by extent of evaluation.

Evaluation Status	Approaches and Findings
Comparatively evaluated interventions	The Kenyan KS trial found responses with gemcitabine but did not demonstrate superiority over bleomycin–vincristine [20]. A Kenyan pilot trial found no significant difference in leg oedema or quality of life when locally made compression bandages were added to KS chemotherapy [21]. A South African cervical cancer trial found better six-month local disease-free survival with added modulated electro-hyperthermia in the overall mixed HIV-status cohort; the association was not statistically significant within its HIV-positive subgroup [22].
Implemented approaches with observational outcomes	Rural Malawian KS care combined protocol-guided chemotherapy, ART and community support; a Mozambican centre introduced pegylated liposomal doxorubicin; Malawian lymphoma services delivered standardised chemotherapy with concurrent ART [29,45,52]. Their outcomes establish delivery in those settings but do not isolate the benefit of each service component.
Proposed approaches/reported facilitators; no intervention-outcome evaluation	Participants and providers recommended closer coordination or colocation of HIV and oncology visits, clearer treatment information, peer and psychosocial support, assistance with transport or insurance, stronger referral pathways, and palliative-care training. These proposals arose from qualitative studies and surveys rather than evaluations of their effects on survival or treatment completion [9,10,24,25,26,27].

**Table 3:** Country-by-cancer evidence map (publication/report counts).

Setting	KS	Cervical	Lymphoma	Breast	Other/Cross-Cancer	Total
Botswana	0	8	1	3	2	14
South Africa	1	2	3	4	0	10
Malawi	2	0	6	0	0	8
Kenya	6	0	0	0	0	6
Tanzania	1	1	0	1	1	4
Uganda	1	1	1	0	1	4
Mozambique	2	0	0	0	0	2
Côte d’Ivoire	0	1	0	0	0	1
Nigeria	0	0	0	0	1	1
Multicountry provider survey *	0	0	0	0	1	1
Other 38 eligible countries	0	0	0	0	0	0
Total	13	13	11	8	6	51

* One report covers Malawi, South Africa, Uganda and Zimbabwe and is counted once. Zimbabwe has no single-country report. Counts include clinical, qualitative and barrier evidence and do not represent unique patient cohorts. Zero means no eligible report, not no cancer care or services. The complete 48-country map is in Supplementary Table S3.

## Data Availability

This review analysed information reported in the cited publications; no new primary patient data were generated. Study-design classifications, critical-appraisal judgments and report-level evidence maps are provided in Supplementary Tables S2–S8.

## Supplementary Materials

The following supporting information can be downloaded at: https://www.mdpi.com/article/10.3390/idr18050122/s1, Table S1: Supplied PubMed strategy, country coverage and search-flow figures; Table S2: Confirmed and potentially overlapping report families; Table S3: Country-by-cancer evidence map for all 48 eligible countries; Table S4: Study design allocation; Table S5: Randomised intervention reports; Table S6: Longitudinal cohort reports; Table S7: Cross-sectional survey reports; Table S8: Qualitative interview reports.

## Abbreviations

ABVD	Doxorubicin, bleomycin, vinblastine and dacarbazine
AIDS	Acquired immunodeficiency syndrome
ART	Antiretroviral therapy
CASP	Critical Appraisal Skills Programme
CD4	Cluster of differentiation 4; used in reference to CD4-positive T-cell counts
CHOP	Cyclophosphamide, doxorubicin, vincristine and prednisone
CI	Confidence interval
EPOCH	Etoposide, prednisone, vincristine, cyclophosphamide and doxorubicin
HIV	Human immunodeficiency virus
KS	Kaposi sarcoma
KSHV	Kaposi sarcoma-associated herpesvirus
NHL	Non-Hodgkin lymphoma
PRESS	Peer Review of Electronic Search Strategies
RNA	Ribonucleic acid
UNAIDS	Joint United Nations Programme on HIV/AIDS
WHO	World Health Organization
EHA	European Haematology Association
ESMO	European Society for Medical Oncology
ASCO	American Society of Clinical Oncology
G-CSF	Granulocyte colony-stimulating factor
JBI	Joanna Briggs Institute
PRISMA-ScR	Preferred Reporting Items for Systematic Reviews and Meta-Analyses extension for Scoping Reviews
SABCHO	South African Breast Cancer and HIV Outcomes
AJOL	African Journals Online
CINAHL	Cumulative Index to Nursing and Allied Health Literature
OSF	Open Science Framework
SSA	Sub-Saharan Africa
