# Supplementary material for: Management of HIV-Associated Cancers in Sub-Saharan Africa: A Scoping Review of Treatment Outcomes, Challenges, and Strategies for Enhancing Care
Source: Infect Dis Rep. 2026 Oct 21;18(5):122. doi: 10.3390/idr18050122 (PMC13704566; doi:10.3390/idr18050122)
Supplement: Supplementary file 1 [file idr-18-00122-s001.zip › idr-4626576-supplementary.pdf]

Supplementary tables

The MEDLINE strategy was developed first and adapted to the indexing and syntax of the other databases.

Table S1A Illustrative search yields and dates

Source	Initial to 17 Aug	Update to 31 Aug	Combined
PubMed/MEDLINE	467	0	467
EBSCO	3671	0	3671
CINAHL	466	0	466
African Journals Online	10	0	10
Google Scholar	998	0	998
Hand Search	26	0	26
Additional/secondary search	0	43	43
ClinicalTrials.gov	109	0	109

Table S1B Country coverage and variant names

The country inventory follows the United Nations M49 grouping of 48 sovereign states in Eastern, Middle, Southern and Western Africa. Current names and frequently encountered former names are searchable. Sudan is not used as a country synonym for South Sudan; the PubMed regional MeSH heading can nevertheless retrieve Sudan, so records found through broader indexing are checked for the actual study setting. Classification source: United Nations Statistics Division, https://unstats.un.org/unsd/methodology/m49/ (accessed 14 October 2026).

M49 subregion	Current country names
Eastern Africa	Burundi; Comoros; Djibouti; Eritrea; Ethiopia; Kenya; Madagascar; Malawi; Mauritius; Mozambique; Rwanda; Seychelles; Somalia; South Sudan; Uganda; United Republic of Tanzania; Zambia; Zimbabwe
Middle Africa	Angola; Cameroon; Central African Republic; Chad; Congo; Democratic Republic of the Congo; Equatorial Guinea; Gabon; Sao Tome and Principe
Southern Africa	Botswana; Eswatini; Lesotho; Namibia; South Africa
Western Africa	Benin; Burkina Faso; Cabo Verde; Cote d'Ivoire; Gambia; Ghana; Guinea; Guinea-Bissau; Liberia; Mali; Mauritania; Niger; Nigeria; Senegal; Sierra Leone; Togo

Additional names in the actual strings: Benin (Dahomey); Botswana (Bechuanaland); Burkina Faso (Upper Volta); Cabo Verde (Cape Verde); Congo (Republic of the Congo, Congo-Brazzaville); Cote d'Ivoire (Côte d'Ivoire, Ivory Coast); Democratic Republic of the Congo (DR Congo, DRC, Congo-Kinshasa, Zaire); Eswatini (Swaziland); Gambia (The Gambia); Ghana (Gold Coast); Guinea-Bissau (Guinea Bissau, Portuguese Guinea); Lesotho (Basutoland); Malawi (Nyasaland); Namibia (South West Africa); Sao Tome and Principe (São Tomé and Príncipe); South Sudan (Southern Sudan); United Republic of Tanzania (Tanzania, Tanganyika); Zambia (Northern Rhodesia); Zimbabwe (Rhodesia, Southern Rhodesia).

PubMed MEDLINE and in-process records

Interface: PubMed Advanced Search. Search all PubMed citations, without imposing medline[sb], so recently supplied or unindexed citations remain eligible. Enter lines P1–P3 as separate history searches. P4 combines them; P5 adds publication dates. The initial P5 upper bound is 2026/08/17; replace it with 2026/08/31 for the update. Export all newly retrieved citations and deduplicate across runs.

P1 HIV "HIV Infections"[Mesh] OR "Acquired Immunodeficiency Syndrome"[Mesh] OR HIV[tiab] OR "HIV-1"[tiab] OR "HIV-2"[tiab] OR AIDS[tiab] OR "human immunodeficiency virus"[tiab] OR "people living with HIV"[tiab] OR "HIV positive"[tiab] OR "HIV-positive"[tiab]

P2 Cancer "Neoplasms"[Mesh] OR "Sarcoma, Kaposi"[Mesh] OR neoplasm*[tiab] OR cancer*[tiab] OR malignan*[tiab] OR tumor*[tiab] OR tumour*[tiab] OR carcinoma*[tiab] OR sarcoma*[tiab] OR lymphoma*[tiab] OR leukemi*[tiab] OR leukaemi*[tiab] OR oncolog*[tiab] OR myeloma*[tiab]

P3 Geography "Africa South of the Sahara"[Mesh] OR "sub-Saharan Africa"[tiab] OR "sub Saharan Africa"[tiab] OR "subsaharan Africa"[tiab] OR "Africa south of the Sahara"[tiab] OR "East Africa"[tiab] OR "Eastern Africa"[tiab] OR "West Africa"[tiab] OR "Western Africa"[tiab] OR "Central Africa"[tiab] OR "Middle Africa"[tiab] OR "Southern Africa"[tiab] OR "Horn of Africa"[tiab] OR Burundi[tiab] OR Comoros[tiab] OR Djibouti[tiab] OR Eritrea[tiab] OR Ethiopia[tiab] OR Kenya[tiab] OR Madagascar[tiab] OR Malawi[tiab] OR Mauritius[tiab] OR Mozambique[tiab] OR Rwanda[tiab] OR Seychelles[tiab] OR Somalia[tiab] OR "South Sudan"[tiab] OR Uganda[tiab] OR "United Republic of Tanzania"[tiab] OR Zambia[tiab] OR Zimbabwe[tiab] OR Angola[tiab] OR Cameroon[tiab] OR "Central African Republic"[tiab] OR Chad[tiab] OR Congo[tiab] OR "Democratic Republic of the Congo"[tiab] OR "Equatorial Guinea"[tiab] OR Gabon[tiab] OR "Sao Tome and Principe"[tiab] OR Botswana[tiab] OR Eswatini[tiab] OR Lesotho[tiab] OR Namibia[tiab] OR "South Africa"[tiab] OR Benin[tiab] OR "Burkina Faso"[tiab] OR "Cabo Verde"[tiab] OR "Cote d'Ivoire"[tiab] OR Gambia[tiab] OR Ghana[tiab] OR Guinea[tiab] OR "Guinea-Bissau"[tiab] OR Liberia[tiab] OR Mali[tiab] OR Mauritania[tiab] OR Niger[tiab] OR Nigeria[tiab] OR Senegal[tiab] OR "Sierra Leone"[tiab] OR Togo[tiab] OR Dahomey[tiab] OR Bechuanaland[tiab] OR "Upper Volta"[tiab] OR "Cape Verde"[tiab] OR "Republic of the Congo"[tiab] OR "Congo-Brazzaville"[tiab] OR "Côte d'Ivoire"[tiab] OR "Ivory Coast"[tiab] OR "DR Congo"[tiab] OR DRC[tiab] OR "Congo-Kinshasa"[tiab] OR Zaire[tiab] OR Swaziland[tiab] OR "The Gambia"[tiab] OR "Gold Coast"[tiab] OR "Guinea Bissau"[tiab] OR "Portuguese Guinea"[tiab] OR Basutoland[tiab] OR Nyasaland[tiab] OR "South West Africa"[tiab] OR "São Tomé and Príncipe"[tiab] OR "Southern Sudan"[tiab] OR Tanzania[tiab] OR Tanganyika[tiab] OR "Northern Rhodesia"[tiab] OR Rhodesia[tiab] OR "Southern Rhodesia"[tiab]

P4 Combine #1 AND #2 AND #3

P5 Date #4 AND 2015/01/01:2026/08/31

Table S2. Confirmed and potentially overlapping report families

Report family	Named reports	Relationship and certainty
Kenyan KS trial	Busakhala 2018; Busakhala 2019 [1,2]	Confirmed: the follow-up identifies the same 70 analysed trial participants.
Botswana curative cervical cohort	Grover 2018; MacDuffie 2021 [3,4]	Confirmed: both describe the same 143 women initiating curative chemoradiation (96 with HIV).
Broader Botswana cervical programmes	Dryden-Peterson 2016; Grover 2018, 2019, 2020, 2022, 2025; MacDuffie 2021; Meghani 2024 [3–10]	Potential/unquantified beyond the confirmed pair above. Recruitment includes shared Botswana referral programmes and intersecting periods (e.g., 2010–2015, 2013–2020 and the 2015–2019 Ipabalele cohort), but entry criteria and time origins differ.
Kamuzu Central Hospital NHL / tumour subsets	Gopal 2016; Kaimila 2017; Zuze 2018; Painschab 2019 [11–14]	Potential overlap within the same lymphoma programme: recruitment windows span 2013–2015, 2013–2016, 2013–2017 and 2013–2018, respectively. Kaimila is HIV-only; plasmablastic and Burkitt reports are subtype subsets.
Kamuzu Central Hospital Hodgkin subsets	Westmoreland 2017; Kaimila 2017; Mponda 2024 [12,15,16]	Potential nested overlap: Westmoreland recruited 2013–2015, Kaimila includes five people with HIV and Hodgkin lymphoma in 2013–2016, and Mponda recruited patients aged ≥15 years during 2013–2021.
Botswana Thabatse breast cohort	Dykstra 2024; Johnson 2024 [17,18]	Confirmed shared parent cohort; exact participant overlap is unquantified. Dykstra uses a survivor quality-of-life subset, while Johnson uses women with HIV and hormone-positive breast cancer for an ART comparison.
South African SABCHO breast cohort	O'Neil 2023; Phakathi 2021 [19,20]	Confirmed shared parent study; exact overlap between analysis subsets is unquantified. Phakathi recruited at two Johannesburg hospitals in 2015–2017; O'Neil used a chemotherapy subset of the wider SABCHO study.

Counts throughout the review are publication-level. This table identifies documented parent cohorts and plausible links. No verified count of unique cohorts or total independent patients can be calculated from the available reports alone.

Table S3. Country-by-cancer evidence map for all 48 eligible countries

Country	KS	Cervical	Lympho ma	Breast	Other / cross-cancer	Single-country reports	Multicountry coverage*
Angola	0	0	0	0	0	0	—
Benin	0	0	0	0	0	0	—
Botswana	0	8	1	3	2	14	—
Burkina Faso	0	0	0	0	0	0	—
Burundi	0	0	0	0	0	0	—
Cabo Verde	0	0	0	0	0	0	—
Cameroon	0	0	0	0	0	0	—
Central African Republic	0	0	0	0	0	0	—
Chad	0	0	0	0	0	0	—
Comoros	0	0	0	0	0	0	—
Congo	0	0	0	0	0	0	—
Côte d'Ivoire	0	1	0	0	0	1	—
Democratic Republic of the Congo	0	0	0	0	0	0	—
Djibouti	0	0	0	0	0	0	—
Equatorial Guinea	0	0	0	0	0	0	—
Eritrea	0	0	0	0	0	0	—
Eswatini	0	0	0	0	0	0	—
Ethiopia	0	0	0	0	0	0	—
Gabon	0	0	0	0	0	0	—
Gambia	0	0	0	0	0	0	—
Ghana	0	0	0	0	0	0	—
Guinea	0	0	0	0	0	0	—
Guinea-Bissau	0	0	0	0	0	0	—
Kenya	6	0	0	0	0	6	—

Country	KS	Cervical	Lymphoma	Breast	Other / cross-cancer	Single-country reports	Multicountry coverage*
Lesotho	0	0	0	0	0	0	—
Liberia	0	0	0	0	0	0	—
Madagascar	0	0	0	0	0	0	—
Malawi	2	0	6	0	0	8	M
Mali	0	0	0	0	0	0	—
Mauritania	0	0	0	0	0	0	—
Mauritius	0	0	0	0	0	0	—
Mozambique	2	0	0	0	0	2	—
Namibia	0	0	0	0	0	0	—
Niger	0	0	0	0	0	0	—
Nigeria	0	0	0	0	1	1	—
Rwanda	0	0	0	0	0	0	—
Sao Tome and Principe	0	0	0	0	0	0	—
Senegal	0	0	0	0	0	0	—
Seychelles	0	0	0	0	0	0	—
Sierra Leone	0	0	0	0	0	0	—
Somalia	0	0	0	0	0	0	—
South Africa	1	2	3	4	0	10	M
South Sudan	0	0	0	0	0	0	—
Togo	0	0	0	0	0	0	—
Uganda	1	1	1	0	1	4	M
United Republic of Tanzania	1	1	0	1	1	4	—
Zambia	0	0	0	0	0	0	—
Zimbabwe	0	0	0	0	0	0	M
Single-country total	13	13	11	8	5	50	One survey*

Country	KS	Cervical	Lymphoma	Breast	Other / cross-cancer	Single-country reports	Multicountry coverage*
Multicountry report*	0	0	0	0	1	1	M in four countries
All reports	13	13	11	8	6	51	Counted once

* M identifies participation in the same single oncology-provider survey covering Malawi, South Africa, Uganda and Zimbabwe; it is not four separate reports or HIV-specific patient-outcome evidence. The multicountry row is counted once in the 51-report total. Zeros indicate no eligible report identified by this review, not absence of malignancy, treatment facilities or services. Report counts include barrier/qualitative evidence and do not measure independent cohorts or service availability.

Critical appraisal of 51 included reports using design specific CASP checklists

Study: Management of HIV-Associated Cancers in Sub-Saharan Africa: A Scoping Review of Treatment Outcomes, Challenges, and Strategies for Enhancing Care

Appraisal approach

The 51 included full texts were grouped by their actual design. Three were randomised intervention reports, 42 longitudinal cohort reports (including retrospective treatment series and a trial follow-up), two cross-sectional surveys, and four qualitative studies. Each report was appraised against the corresponding 2024 Critical Appraisal Skills Programme (CASP) checklist. A longitudinal treatment series was assigned the cohort form because participants were followed for outcomes; no separate CASP case-series checklist was used.

Y = yes, **N** = no, and **CT** = cannot tell from the reported information or the available estimate. CT does not establish that a method was absent. **Item** judgements and paper-specific considerations are reported without a **numerical** total or quality-based exclusion. Ratings address each report's stated question, not proof of a causal effect. Mixed HIV/non-HIV samples remain included, but pooled estimates must not be reported as HIV-only outcomes.

CASP sources (2024): https://casp-uk.net/casp-checklists/CASP-checklist-randomised-controlled-trials-RCT-2024.pdf ; https://casp-uk.net/casp-checklists/CASP-checklist-cohort-study-2024.pdf; https://casp-uk.net/casp-checklists/CASP-checklist-cross-sectional-study-2024.pdf; https://casp-uk.net/casp-checklists/CASP-checklist-qualitative-2024.pdf (accessed 14 October 2026). Item **names below are abbreviated paraphrases of these forms.**

Table S4 Study design allocation

Design group	n	CASP form used	Basis for classification
Randomised intervention	3	Randomised controlled trial	Assignment to treatment or adjunct was randomised.
Longitudinal cohort	42	Cohort study	Outcomes were followed over time, prospectively or from records; includes descriptive single-arm series and trial follow-up.

Design group	n	CASP form used	Basis for classification
Cross-sectional survey	2	Descriptive/cross-sectional study	Single-time-point patient or provider questionnaires.
Qualitative interview	4	Qualitative research	Interview data analysed for patient or provider experiences.
Total	51		One checklist form per report, matched to its primary analysed design.

Checklist item guide

Randomised trials: R1 focused question; R2 random sequence and concealment; R3 participant accounting and assigned-group analysis; R4a participants masked; R4b intervention staff masked; R4c outcome assessment masked; R5 baseline balance; R6 otherwise comparable care; R7 complete effects reporting; R8 precision; R9 benefit, harm and cost balance; R10 applicability; R11 added value relative to current care.

Longitudinal cohorts: C1 focused question; C2 appropriate cohort recruitment; C3 exposure measurement; C4 outcome measurement; C5a relevant confounders identified; C5b confounders addressed; C6a follow-up completeness; C6b follow-up duration; C7 results presented; C8 precision; C9 credibility of inference; C10 applicability; C11 fit with other evidence; C12 implications for care.

Cross-sectional surveys: X1 focused question; X2 appropriate design; X3 recruitment; X4 valid measurement; X5 data collection; X6 adequate sample; X7 results presented; X8 analytical rigour; X9 clear findings; X10 applicability; X11 value.

Qualitative studies: Q1 clear aims; Q2 qualitative approach; Q3 appropriate design; Q4 participant recruitment; Q5 data collection; Q6 researcher-participant reflexivity; Q7 ethics; Q8 analytical rigour; Q9 findings supported; Q10 value and transferability.

Table S5 Randomised intervention reports

Report	R1	R2	R3	R4a	R4b	R4c	R5	R6	R7	R8	R9	R10	R11
Busakhala et al., 2018 [1]	Y	CT	N	N	N	CT	Y	Y	CT	CT	CT	CT	CT
Chang et al., 2022 [21]	Y	Y	N	N	N	Y	Y	CT	CT	Y	CT	CT	CT
Minnaar et al., 2019 [22]	Y	Y	CT	N	N	Y	Y	Y	Y	Y	CT	CT	CT

Interpretive notes for Table S5

Report	Design	Evidence supporting the appraisal and main caution
Busakhala et al., 2018 [1]	Randomised phase II trial	Biopsy-confirmed KS and a prospective comparison; allocation sequence and concealment are insufficiently described, which is not proof that concealment was absent. The flow diagram reports 83 randomised (40 gemcitabine; 43 bleomycin–vincristine) but 70 analysed (36 and 34), whereas the narrative describes a 70-person trial. Post-randomisation exclusions and incomplete assigned-group accounting limit interpretation. Participant/staff and response-assessor masking were not sufficiently documented; do not infer assessor masking. Responses were assessed after three cycles; the 2019 report follows the same analysed population.
Chang et al., 2022 [21]	Randomised compression pilot	Computer-generated blocks, opaque sealed envelopes and a masked assessor strengthen allocation and measurement. Participants and providers were unmasked. Thirty were randomised; one was subsequently deemed ineligible and 25 contributed week-14 outcomes among the 29 eligible participants. Missing follow-up limits a complete assigned-group comparison. Small numbers, variable chemotherapy exposure and interruptions in chemotherapy availability further limit precision and generalisability; null findings do not establish equivalence.
Minnaar et al., 2019 [22]	Phase III randomised trial, interim endpoint	HIV-stratified secure online allocation and masked reporting clinicians/image readers support the comparison; participants and treatment staff were unmasked. Of 210 randomised, 202 were evaluable at six months. Local control was assessed among survivors with available evaluation; the local disease-free endpoint used a different denominator, and neither should be presented as complete follow-up of all 210. Conditioning on survival/assessment can bias local-control comparisons. Overall mixed-cohort findings did not establish a statistically significant benefit in the HIV-positive subgroup, and six-month outcomes do not establish long-term survival benefit.

Table S6 Longitudinal cohort reports

The 42 reports include single-arm treatment cohorts, comparative observational cohorts, and follow-up of participants from a previous randomised trial. C5b and C9 concern the comparison or causal interpretation relevant to this review.

Report	C1	C2	C3	C4	C5a	C5b	C6a	C6b	C7	C8	C9	C10	C11	C12
Adinani et al., 2021 [23]	Y	CT	Y	CT	N	N	Y	Y	Y	CT	CT	CT	CT	CT
Boni et al., 2023 [24]	Y	CT	Y	Y	Y	Y	CT	Y	Y	Y	CT	CT	CT	Y
Busakhala et al., 2019 [2]	Y	CT	Y	Y	Y	Y	CT	Y	Y	Y	CT	CT	CT	CT
Coldiron et al., 2021 [25]	Y	CT	Y	Y	Y	Y	CT	Y	Y	Y	CT	CT	CT	Y
Dryden-Peterson et al., 2016 [5]	Y	Y	CT	Y	Y	Y	Y	Y	Y	Y	CT	CT	CT	Y
Dykstra et al., 2024 [17]	Y	CT	Y	Y	Y	Y	CT	Y	Y	Y	CT	CT	CT	Y
Fardhdiani et al., 2018 [26]	Y	CT	Y	CT	Y	Y	N	Y	Y	Y	N	CT	CT	Y
Freeman et al., 2020 [27]	Y	Y	CT	Y	Y	Y	CT	Y	Y	Y	Y	Y	CT	Y
Gopal et al., 2016 [11]	Y	CT	Y	Y	Y	Y	Y	Y	Y	Y	CT	CT	CT	Y
Grover et al., 2018 [3]	Y	CT	Y	Y	Y	Y	CT	Y	Y	Y	CT	CT	CT	Y
Grover et al., 2019 [7]	Y	CT	Y	Y	Y	Y	CT	Y	Y	Y	CT	CT	CT	Y
Grover et al., 2020 [8]	Y	CT	CT	Y	Y	Y	CT	Y	Y	Y	CT	CT	CT	CT
Grover et al., 2022 [6]	Y	CT	Y	Y	Y	Y	CT	Y	Y	Y	CT	CT	CT	Y
Grover et al., 2025 [9]	Y	CT	Y	Y	Y	Y	CT	Y	Y	Y	CT	CT	CT	Y
Herce et al., 2015 [28]	Y	CT	Y	Y	CT	N	Y	Y	Y	Y	CT	CT	CT	Y
Johnson et al., 2024 [18]	Y	CT	Y	Y	Y	CT	CT	Y	Y	Y	CT	CT	CT	CT
Kaimila et al., 2017 [12]	Y	CT	Y	Y	CT	N	CT	Y	CT	CT	CT	CT	CT	CT
Kavuma et al., 2022 [29]	Y	N	Y	Y	Y	N	CT	Y	Y	CT	CT	CT	CT	CT
Langenhoven et al., 2016 [30]	Y	CT	CT	Y	Y	Y	CT	Y	Y	CT	CT	CT	CT	CT
Low et al., 2019 [31]	Y	CT	CT	CT	CT	N	CT	Y	Y	CT	CT	CT	CT	Y
MacDuffie et al., 2021 [4]	Y	CT	Y	Y	Y	Y	CT	Y	Y	Y	CT	CT	CT	Y
Macken et al., 2018 [32]	Y	CT	Y	Y	Y	CT	N	Y	Y	Y	CT	CT	CT	CT
McGinnis et al., 2021 [33]	Y	CT	Y	Y	Y	Y	CT	Y	Y	Y	CT	CT	CT	Y
McLigeyo et al., 2022 [34]	Y	CT	CT	CT	N	N	CT	CT	Y	CT	CT	CT	CT	CT

Report	C1	C2	C3	C4	C5a	C5b	C6a	C6b	C7	C8	C9	C10	C11	C12
Mduma et al., 2023 [35]	Y	CT	Y	Y	Y	Y	CT	Y	Y	Y	CT	CT	CT	CT
Meghani et al., 2024 [10]	Y	CT	Y	Y	Y	Y	CT	Y	Y	Y	CT	CT	CT	Y
Moahi et al., 2022 [36]	Y	CT	Y	CT	Y	Y	Y	Y	Y	Y	CT	CT	CT	Y
Mponda et al., 2024 [16]	Y	CT	Y	Y	Y	CT	CT	Y	Y	CT	CT	CT	CT	Y
Mrema et al., 2023 [37]	Y	CT	Y	CT	Y	Y	CT	Y	Y	Y	CT	CT	CT	Y
Musekwa et al., 2020 [38]	Y	CT	Y	Y	CT	N	CT	Y	Y	CT	CT	CT	CT	CT
O'Neil et al., 2023 [19]	Y	N	Y	Y	Y	Y	CT	Y	Y	Y	CT	CT	CT	Y
Okello et al., 2020 [39]	Y	CT	Y	CT	Y	CT	CT	Y	Y	Y	CT	CT	CT	CT
Painschab et al., 2019 [14]	Y	CT	Y	Y	Y	CT	Y	Y	Y	Y	CT	CT	CT	Y
Phakathi et al., 2016 [40]	Y	CT	Y	Y	Y	CT	CT	CT	Y	CT	CT	CT	CT	CT
Phakathi et al., 2021 [20]	Y	CT	Y	Y	Y	Y	Y	Y	Y	Y	CT	CT	CT	Y
Phipps et al., 2023 [41]	Y	CT	Y	Y	Y	CT	CT	Y	Y	Y	CT	CT	CT	Y
Rapiti et al., 2022 [42]	Y	CT	Y	Y	CT	N	CT	Y	Y	CT	CT	CT	CT	CT
Sengayi et al., 2017 [43]	Y	CT	Y	Y	Y	Y	CT	Y	Y	Y	CT	CT	CT	Y
Turdo et al., 2022 [44]	Y	N	CT	Y	Y	Y	CT	Y	Y	Y	CT	N	CT	CT
Vogt et al., 2024 [45]	Y	CT	Y	Y	Y	CT	CT	Y	Y	Y	CT	CT	CT	Y
Westmoreland et al., 2017 [15]	Y	CT	Y	Y	Y	N	CT	Y	Y	Y	CT	CT	CT	CT
Zuze et al., 2018 [13]	Y	CT	Y	Y	CT	N	Y	CT	Y	Y	CT	CT	CT	CT

Table S6 Longitudinal cohort evidence notes

Report	Design	Evidence supporting the appraisal and main caution
Adinani et al., 2021 [23]	Retrospective paediatric treatment cohort	Single-centre paclitaxel series with no reported loss to follow-up. Most KS diagnoses lacked histological confirmation, toxicity surveillance was mainly clinical, and the small treated-only sample does not estimate comparative efficacy.
Boni et al., 2023 [24]	Prospective cervical cancer cohort	Consecutive recruitment, HIV stratification and adjusted access/survival analyses are strengths. Enrolment required reaching participating cancer centres; survival and care-access estimates omit women who never reached diagnosis or these centres.
Busakhala et al., 2019 [2]	Post-trial three-year follow-up cohort	Survival follow-up of the same 70 analysed patients as Busakhala 2018, with Cox analyses and a three-year endpoint. Original post-randomisation exclusions and only ten deaths constrain arm comparisons; this report is not an independent patient sample.
Coldiron et al., 2021 [25]	Prospective single-arm treatment cohort	Prospective treatment and 24-month follow-up with clinical response, toxicity and HIV variables. Patients with poor performance status were excluded, 15/116 were lost, and the single-arm design cannot separate PLD benefit from selection and concurrent ART.
Dryden-Peterson et al., 2016 [5]	Prospective comparative cervical cancer cohort	Sequential enrolment, objective treatment records, active vital-status ascertainment and inverse-probability weighting strengthen analysis. HIV status was unavailable in part of the enrolled cohort and treatment was often incomplete; residual differences remain possible.
Dykstra et al., 2024 [17]	Prospective survivor quality-of-life cohort	Repeated validated SF-8 measurement and weighting support the comparison among survivors. The analysis retained 298/603 enrolled women and excluded 137 who died before the later assessment; conclusions concern quality-of-life change in survivors only.
Fardhdiani et al., 2018 [26]	Retrospective Kaposi sarcoma programme cohort	Large routine-care cohort and recorded ART/chemotherapy exposure. Among chemotherapy starters, 43% were lost to follow-up; recorded deaths therefore substantially underascertain mortality, and fragmented routine data weaken outcome classification.
Freeman et al., 2020 [27]	Retrospective multiclinic inception cohort	An HIV primary-care network identified new KS diagnoses and treated death as a competing event for chemotherapy initiation. Charts were available for 588/674 patients; retrospective KS severity and missing files could bias the estimated treatment gap.

Report	Design	Evidence supporting the appraisal and main caution
Gopal et al., 2016 [11]	Prospective lymphoma treatment cohort	Pathology-confirmed lymphoma, standardized CHOP, adjusted prognostic analyses and known vital status in all 58 patients aid interpretation. Referral selection, heterogeneous NHL subtypes and small HIV-stratified samples constrain causal comparisons.
Grover et al., 2018 [3]	Prospective curative-CRT cohort	Prospective treatment and toxicity ascertainment with stage/treatment adjustment. Only 143 of 182 treated women initiating curative CRT entered the analysis; all women with HIV were on ART, limiting inference for untreated or poorly controlled HIV.
Grover et al., 2019 [7]	Prospective cervical cancer access cohort	The treatment-initiation denominator includes curative and non-curative patients (519), and logistic models address stage and haemoglobin. Referral-centre enrolment and clinician selection of treatment intent may leave unmeasured access differences.

Table S6 Longitudinal cohort evidence notes continued

Report	Design	Evidence supporting the appraisal and main caution
Grover et al., 2020 [8]	Prospective HIV-immunology cervical cohort	Adjusted survival models examine historical and current CD4 measures. Nadir CD4 was analysed in 231 women, change in CD4 in only 139; nonregular measurements, incomplete viral-load/ART adherence data and shared Botswana recruitment limit inference.
Grover et al., 2022 [6]	Prospective cervical cancer inception cohort	A large cohort of 1,043 diagnosed patients provides stage- and HIV-stratified outcomes and adjusted analyses. Referral-centre recruitment, limited staging imaging and overlap with other Botswana cervical reports constrain generalisation and independence.
Grover et al., 2025 [9]	Prospective curative-CRT long-term cohort	Up-to-five-year follow-up with stage-adjusted overall survival. Selection of curative CRT recipients and incomplete late toxicity/recurrence ascertainment mean apparent HIV parity applies to this treated group and this endpoint.
Herce et al., 2015 [28]	Retrospective integrated-care treatment cohort	Protocol-guided chemotherapy, ART and retention were documented in a rural programme, with few lost at 12 months. Entry required receiving at least one chemotherapy cycle, and no contemporaneous comparator tested the effect of integrated care.
Johnson et al., 2024 [18]	Retrospective ART-exposure breast cancer cohort	The comparison isolates efavirenz exposure within women with HIV and ER-positive breast cancer. A small sample, wide survival confidence intervals and limited adjustment leave ART-regimen effects highly uncertain.
Kaimila et al., 2017 [12]	Prospective HIV lymphoproliferative cohort	Serial CD4 and HIV-RNA testing during concurrent ART/chemotherapy is a strength. The 72-person sample includes non-lymphoma lymphoproliferative disorders; pooled survival is not a lymphoma-specific estimate and small subgroups limit inference.
Kavuma et al., 2022 [29]	Retrospective completed-treatment cohort	Radiotherapy fractionation and HIV status were documented, with five-year survival and a separate 24-month response assessment. Inclusion required completion of external beam radiation and brachytherapy; unadjusted group comparisons omit patients who could not complete treatment.
Langenhoven et al., 2016 [30]	Retrospective matched breast treatment cohort	Age/ethnicity matching supports a treatment-completion comparison. HIV status was untested in 135/586 women and only 31 were HIV-positive; chart-based toxicity and residual confounding limit subgroup conclusions.

Report	Design	Evidence supporting the appraisal and main caution
Low et al., 2019 [31]	Prospective interview and follow-up cohort	Structured interviews captured patient-reported delays/barriers and a one-year reassessment. Recruitment at the cancer institute misses people who never arrived; recalled prediagnostic dates and no HIV-negative comparator constrain comparisons.
MacDuffie et al., 2021 [4]	Five-year curative-CRT follow-up cohort	Longer-term vital-status follow-up of the same 143 women as Grover 2018, with multivariable analyses. All HIV-positive women were receiving ART, and only curative-CRT starters are represented; this is not an independent cohort.
Macken et al., 2018 [32]	Paediatric treatment and historical-comparison cohort	A defined paediatric KS regimen and serial survival are described. Loss to follow-up increased to about 21% by 24 months, while the comparison with a previously reported treatment group lacks contemporaneous allocation.

Table S6 Longitudinal cohort evidence notes continued

Report	Design	Evidence supporting the appraisal and main caution
McGinnis et al., 2021 [33]	Prospective head-and-neck cancer cohort	Prospective registration and survival/toxicity models provide clinical detail. Clinic enrolment, unequal treatments and a modest HIV subgroup (59/149) limit interpretation of an apparent null HIV association.
McLigeyo et al., 2022 [34]	Retrospective Kaposi sarcoma treatment series	Records from 11 hubs identified 95 suspected KS cases, of which 67 were histologically confirmed. Missing KS stage and ART-start dates, retrospective response recording and no comparator constrain treatment-effect claims.
Mduma et al., 2023 [35]	Retrospective anal cancer treatment cohort	Treatment and at least two-year outcomes were retrospectively evaluated in 59 patients. The small HIV stratum and centre-based treated denominator make pooled remission/survival inappropriate as HIV-only outcomes; comparative estimates are imprecise.
Meghani et al., 2024 [10]	Prospective cervical cancer treatment cohort	Large enrolled Botswana cohort and multivariable models stratify outcomes by treatment received. Differences between curative CRT and radiation-only groups reflect treatment selection; two-year follow-up and shared Botswana recruitment constrain interpretation.
Moahi et al., 2022 [36]	Prospective Hodgkin lymphoma cohort	Prospective HIV testing, quarterly follow-up and no lost subjects support vital-status comparisons. Therapy/intent data were incomplete and only 78 treated patients were enrolled, making weighted HIV estimates susceptible to residual confounding.
Mponda et al., 2024 [16]	Prospective single-centre Hodgkin cohort	Pathology-confirmed HL, defined ABVD protocol, toxicity and microcosting strengthen description. The cohort comprised 38 patients, only 11 with HIV; estimates by HIV and treatment-related risk are imprecise.
Mrema et al., 2023 [37]	Prospective early-response cervical cohort	Prospective HIV testing, RECIST-based three-month assessment and adjusted logistic models support early response findings. Analysis was restricted to curative-intent treated patients; ultrasound/clinical assessment and short follow-up do not establish durable control.
Musekwa et al., 2020 [38]	Retrospective Burkitt lymphoma survival cohort	Registry/hospital records gave at least 24-month outcome opportunities. Five of 73 diagnosed cases were omitted from detailed analysis; variable regimens, differences between adult and paediatric cases and limited adjustment restrict comparisons by HIV.

Report	Design	Evidence supporting the appraisal and main caution
O'Neil et al., 2023 [19]	Prospective breast chemotherapy cohort analysis	Recorded drugs/doses, laboratory toxicity and multivariable analyses support an exposure-specific comparison. Selection required at least two chemotherapy doses, excluding very early discontinuation and limiting inference about completion in all eligible women.
Okello et al., 2020 [39]	Retrospective nonrandomised regimen cohort	Chemotherapy regimens and one-year survival were recorded, with CIs and prognostic models. DA-EPOCH was received by only 12 versus 108 on CHOP; treatment assignment and adverse-event capture from charts were not comparably controlled.

Table S6 Longitudinal cohort evidence notes continued

Report	Design	Evidence supporting the appraisal and main caution
Painschab et al., 2019 [14]	Prospective Burkitt lymphoma treatment cohort	Prospective pathology and known vital status for all participants strengthen survival estimates. CHOP-to-EPOCH treatment changed by calendar period; size and heterogeneity preclude attributing outcome differences to regimen alone.
Phakathi et al., 2016 [40]	Prospective breast surgery/treatment cohort	Prospective surgical enrolment and 30-day complication assessment. Only 31 of 160 women had HIV, and short surgical follow-up plus limited confounder control constrain comparisons of later cancer treatment.
Phakathi et al., 2021 [20]	Prospective breast cancer survival cohort	Large newly diagnosed cohort (1,019), HIV-stratified outcomes and adjusted survival analysis, with reported follow-up loss of 3.5%. Referral-centre selection and unavailable cancer-specific cause of death limit attribution of excess mortality to breast cancer.
Phipps et al., 2023 [41]	Prospective Kaposi sarcoma cohort	New KS presentations and HIV/KSHV biomarkers were followed for one year. Treatment varied in routine care, and the HIV-positive subgroup (166/200) differs clinically from HIV-negative endemic KS; adjusted HIV-versus-non-HIV inference is limited.
Rapiti et al., 2022 [42]	Retrospective plasmablastic lymphoma series	Twelve-year chart review of 26 HIV-associated PBL cases provides tumour and regimen detail. Small numbers, heterogeneous salvage therapy and some follow-up loss make comparative treatment estimates unstable.
Sengayi et al., 2017 [43]	Retrospective Kaposi sarcoma survival cohort	ART/treatment information and adjusted calendar-period survival models are available for 357 tertiary-care patients. Unrecorded changes in referral, supportive care and chemotherapy over the two ART eras could explain part of the observed difference.
Turdo et al., 2022 [44]	Retrospective insurance-claims cervical cohort	Claims analysis includes only insured women starting cancer treatment within 180 days; it cannot estimate population-level access. HIV/histology/stage classification relied partly on claims and imputation, and private-sector treatment patterns have limited transferability.
Vogt et al., 2024 [45]	Prospective Hodgkin lymphoma cohort	Pathology review and prospective vital-status follow-up capture early mortality. Two-centre, 47-patient cohort with only 16 HIV-negative comparators; stepwise multivariable estimates are vulnerable to sparse events and treatment differences.

Report	Design	Evidence supporting the appraisal and main caution
Westmoreland et al., 2017 [15]	Prospective mixed-HIV Hodgkin cohort	Pathology/EBV testing and standardized treatment support description of 31 confirmed cases. Limited size precluded adjusted HIV comparisons, and specialist referral may omit patients dying before presentation.
Zuze et al., 2018 [13]	Prospective plasmablastic lymphoma subcohort	All 12 pathology-confirmed patients had known vital status, but only six had HIV; survival CIs are wide and short follow-up limits confidence in HIV subgroup and regimen comparisons. This subgroup may overlap the Malawi lymphoma programme.

Table S7 Cross-sectional survey reports

Report	X1	X2	X3	X4	X5	X6	X7	X8	X9	X10	X11	Appraisal basis and main caution
Bhatia et al., 2018 [46]	Y	Y	CT	CT	CT	Y	Y	CT	Y	CT	Y	A 214-patient survey identifies intervals and barriers, including HIV-stratified characteristics. Convenience recruitment among patients reaching oncology, recalled symptom dates and treatment information for only part of the sample limit representative, precise delay estimates.
Montano et al., 2025 [47]	Y	Y	Y	CT	Y	Y	Y	Y	Y	CT	Y	Staff lists and >90% response within eligible job titles support recruitment; 195 responses span four tertiary centres. The study-team questionnaire measures reported beliefs and hypothetical feasibility, not observed cancer outcomes or implementation effects.

Table S8 Qualitative interview reports

Report	Q1	Q2	Q3	Q4	Q5	Q6	Q7	Q8	Q9	Q10	Appraisal basis and main caution
Idemili-Aronu et al., 2026 [48]	Y	Y	Y	Y	Y	Y	Y	Y	Y	Y	Purposively recruited providers across Nigeria, recorded interviews, iterative saturation review, member checks, an audit trail and reflexive journal strengthen the thematic account. Themes represent provider perceptions rather than measured palliative-care effects.
Martei et al., 2023 [49]	Y	Y	Y	Y	Y	CT	Y	Y	Y	CT	Purposeful high-/low-treatment-fidelity selection, recorded interviews, saturation and double coding support the themes. Only 10/30 interviewees had HIV; HIV-specific insights come from that smaller group, with limited explicit interviewer reflexivity.
McMahon et al., 2022 [50]	Y	Y	Y	Y	Y	CT	Y	Y	Y	Y	Fifty-seven patients were purposively sampled across chemotherapy initiation/completion groups; semistructured interviews and consensus coding support barrier themes. The author's influence on interviews is incompletely discussed and centre-linked recruitment may miss non-presenters.
Nyagabona et al., 2025 [51]	Y	Y	Y	Y	Y	Y	Y	Y	Y	Y	Purposive interviews with 11 women with HIV/breast cancer and 10 providers, reflexive memoing, iterative coding and ethics procedures support the proposed care model. A 21-person purposive sample cannot estimate strategy effectiveness or prevalence.

References

1. Busakhala NW, Waako PJ, Strother MR, Keter AK, Kigen GK, Asirwa FC, et al. Randomized Phase IIA Trial of Gemcitabine Compared With Bleomycin Plus Vincristine for Treatment of Kaposi's Sarcoma in Patients on Combination Antiretroviral Therapy in Western Kenya. J Glob Oncol. 2018;4:1–9. doi:10.1200/JGO.17.00077

2. Busakhala N, Kigen G, Waako P, Strother RM, Chite F, Sr PL. Three year survival among patients with aids-related Kaposi sarcoma treated with chemotherapy and combination antiretroviral therapy at Moi teaching and referral hospital, Kenya. Infect Agent Cancer. 2019;14(1):24. doi:10.1186/s13027-019-0242-9

3. Grover S, Bvochora-Nsingo M, Yeager A, Chiyapo S, Bhatia R, MacDuffie E, et al. Impact of Human Immunodeficiency Virus Infection on Survival and Acute Toxicities From Chemoradiation Therapy for Cervical Cancer Patients in a Limited-Resource Setting. Int J Radiat Oncol Biol Phys. 2018;101(1):201–10. doi:10.1016/j.ijrobp.2018.01.067

4. MacDuffie E, Bvochora-Nsingo M, Chiyapo S, Balang D, Chambers A, George JM, et al. Five-year overall survival following chemoradiation therapy for locally advanced cervical carcinoma in women living with and without HIV infection in Botswana. Infect Agent Cancer. 2021;16(1):55. doi:10.1186/s13027-021-00389-w

5. Dryden-Peterson S, Bvochora-Nsingo M, Suneja G, Efstathiou JA, Grover S, Chiyapo S, et al. HIV Infection and Survival Among Women With Cervical Cancer. J Clin Oncol. 2016;34(31):3749–57. doi:10.1200/JCO.2016.67.9613

6. Grover S, George J, Tuli S, Lichter K, Bhatia R, Monare B, et al. Stage and outcomes of invasive cervical cancer patients in Botswana: A prospective cohort study from 2013 to 2020. Gynecol Oncol Rep. 2022;44:101094. doi:10.1016/j.gore.2022.101094

7. Grover S, MacDuffie E, Wang Q, Bvochora-Nsingo M, Bhatia R, Balang D, et al. HIV infection is not associated with initiation of curative treatment in women with cervical cancer in Botswana. Cancer. 2019;125(10):1645–53. doi:10.1002/cncr.31972

8. Grover S, Mehta P, Wang Q, Bhatia R, Bvochora-Nsingo M, Davey S, et al. Association between CD4 count and chemoradiation therapy outcomes among cervical cancer patients with HIV. J Acquir Immune Defic Syndr. 2020;85(2):201–8. doi:10.1097/QAI.0000000000002420

9. Grover S, Bhatia R, Zhang S, Khan S, Bvochora-Nsingo M, Chiyapo S, et al. Cervical cancer treatment outcomes and survival in Botswana by human immunodeficiency virus status: Ipabalele study results. JNCI Cancer Spectr. 2025. doi:10.1093/jncics/pkaf045

10. Meghani K, Puri P, Bazzett-Matabele L, Vuylsteke P, Luckett R, Monare B, et al. Significance of HIV status in cervical cancer patients receiving curative chemoradiation therapy, definitive radiation alone, or palliative radiation in Botswana. Cancer. 2024;130(14):2462–71. doi:10.1002/cncr.35289

11. Gopal S, Fedoriw Y, Kaimila B, Montgomery ND, Kasonkanji E, Moses A, et al. CHOP Chemotherapy for Aggressive Non-Hodgkin Lymphoma with and without HIV in the Antiretroviral Therapy Era in Malawi. PLOS ONE. 2016;11(3):e0150445. doi:10.1371/journal.pone.0150445

12. Kaimila B, van der Gronde T, Kasonkanji E, Fox P, Chikasema M, Tewete B, et al. CD4 Count and HIV RNA Trends for HIV-Associated Lymphoproliferative Disorders in Malawi. AIDS Res Hum Retroviruses. 2017;33(10):1045–7. doi:10.1089/aid.2017.0081

13. Zuze T, Painschab MS, Seguin R, Kudowa E, Kaimila B, Kasonkanji E, et al. Plasmablastic lymphoma in Malawi. Infect Agent Cancer. 2018;13(1):22. doi:10.1186/s13027-018-0195-4

14. Painschab MS, Westmoreland KD, Kasonkanji E, Zuze T, Kaimila B, Waswa P, et al. Prospective study of Burkitt lymphoma treatment in adolescents and adults in Malawi. Blood Adv. 2019;3(4):612–20. doi:10.1182/bloodadvances.2018029199

15. Westmoreland KD, Stanley CC, Montgomery ND, Kaimila B, Kasonkanji E, El-Mallawany NK, et al. Hodgkin lymphoma, HIV, and Epstein–Barr virus in Malawi: Longitudinal results from the Kamuzu Central Hospital Lymphoma study. Pediatr Blood Cancer. 2017;64(5):e26302. doi:10.1002/pbc.26302

16. Mponda M, Kudowa E, Craven DM, Eastburg LC, Chikasema M, Kasonkanji E, et al. Safety, efficacy, and affordability of ABVD for Hodgkin lymphoma in Malawi: a prospective cohort study. eClinicalMedicine. 2024;69:102480. doi:10.1016/j.eclinm.2024.102480

17. Dykstra MP, Sadigh KS, Nkele I, Bvochora-Nsingo M, Martei YM, Wester J, et al. Quality of Life Gain Following Treatment Among Breast Cancer Survivors with and without HIV. JCO Glob Oncol. 2024;10:e2400110. doi:10.1200/GO.24.00110

18. Johnson AT, Ntloedibe T, Reyes JEM, Matshaba MS, Dryden-Peterson SL, Chiao EY. Impact of efavirenz on hormone-positive breast cancer survival in women living with HIV. AIDS. 2024;38(9):1439–42. doi:10.1097/QAD.0000000000003912

19. O'Neil DS, Ayeni OA, Farrow HA, Chen WC, Demetriou G, Buccimazza I, et al. The Impact of HIV Infection on Neoadjuvant and Adjuvant Chemotherapy Relative Dose Intensity in South African Patients with Breast Cancer. The Oncologist. 2023;28(10):e921–9. doi:10.1093/oncolo/oyad056

20. Phakathi B, Nietz S, Cubasch H, Dickens C, Dix-Peek T, Joffe M, et al. Survival of south african women with breast cancer receiving anti-retroviral therapy for HIV. The Breast. 2021;59:27–36. doi:10.1016/j.breast.2021.05.014

21. Chang AY, Karwa R, Odhiambo H, Were P, Fletcher SL, Tonui EC, et al. Compression Therapy for HIV-Associated Kaposi Sarcoma Leg Lymphedema: Results of the Kenyan Improvised Compression for Kaposi Sarcoma Randomized Controlled Trial. JCO Glob Oncol. 2022;8:e2100329. doi:10.1200/GO.21.00329

22. Minnaar CA, Kotzen JA, Ayeni OA, Naidoo T, Tunmer M, Sharma V, et al. The effect of modulated electro-hyperthermia on local disease control in HIV-positive and -negative cervical cancer women in South Africa: Early results from a phase III randomised controlled trial. PLOS ONE. 2019;14(6):e0217894. doi:10.1371/journal.pone.0217894

23. Adinani H, Campbell L, El-Mallawany NK, Slone J, Mehta P, Bacha J. Use of Paclitaxel to Successfully Treat Children, Adolescents, and Young Adults with Kaposi Sarcoma in Southwestern Tanzania. Children. 2021;8(4):275. doi:10.3390/children8040275

24. BONI SP, HORO A, DIDI-KOUKO-COULIBALY J, TANON A, TCHOUNGA BK, COFFIE PA, et al. Impact of HIV infection on access to cancer care and survival among women with invasive cervical cancer in Côte d'Ivoire: A prospective cohort study. Int J Gynecol Obstet. 2023;163:392–401. doi:10.1002/ijgo.14925

25. Coldiron ME, Zamudio AGG, Manuel R, Luciano G, Rusch B, Ciglenecki I, et al. Outcomes of AIDS-associated Kaposi sarcoma in Mozambique after treatment with pegylated liposomal doxorubicin. Infect Agent Cancer. 2021;16(1):2. doi:10.1186/s13027-020-00341-4

26. Fardhdiani V, Molfino L, Zamudio AG, Manuel R, Luciano G, Ciglenecki I, et al. HIV-associated Kaposi's sarcoma in Maputo, Mozambique: outcomes in a specialized treatment center, 2010–2015. Infect Agent Cancer. 2018;13(1):5. doi:10.1186/s13027-018-0177-6

27. Freeman EE, Busakhala N, Regan S, Asirwa FC, Wenger M, Seth D, et al. Real-world use of chemotherapy for Kaposi's sarcoma in a large community-based HIV primary care system in Kenya. BMC Cancer. 2020;20(1):71. doi:10.1186/s12885-019-6506-3

28. Herce ME, Kalanga N, Wroe EB, Keck JW, Chingoli F, Tengatenga L, et al. Excellent clinical outcomes and retention in care for adults with HIV-associated Kaposi sarcoma treated with systemic chemotherapy and integrated antiretroviral therapy in rural Malawi. J Int AIDS Soc. 2015;18(1):19929. doi:10.7448/IAS.18.1.19929

29. Kavuma A, Luutu I, Kibudde S, Kanyike D. A Retrospective Analysis of the Impact of HIV Infection on Outcomes of Locally Advanced Cervical Cancers Treated With Either Conventional or Hypofractionated Radiotherapy: The Uganda Experience. JCO Glob Oncol. 2022;8:e2100360. doi:10.1200/GO.21.00360

30. Langenhoven L, Barnardt P, Neugut AI, Jacobson JS. Phenotype and Treatment of Breast Cancer in HIV-Positive and -Negative Women in Cape Town, South Africa. J Glob Oncol. 2016;2(5):284–91. doi:10.1200/JGO.2015.002451

31. Low DH, Phipps W, Orem J, Casper C, Ignacio RAB. Engagement in HIV Care and Access to Cancer Treatment Among Patients With HIV-Associated Malignancies in Uganda. J Glob Oncol. 2019;5:1–8. doi:10.1200/JGO.18.00187

32. Macken M, Depani S, Dale H, Israels T, Chagaluka G, Moyo D, et al. Triple therapy of vincristine, bleomycin and etoposide for children with Kaposi sarcoma: Results of a study in Malawian children. Pediatr Blood Cancer. 2018. doi:10.1002/pbc.26841

33. McGinnis GJ, Ning MS, Bvochora-Nsingo M, Chiyapo S, Balang D, Ralefala T, et al. Management of head and neck cancers with or without comorbid HIV infection in Botswana. The Laryngoscope. 2021;131(5):E1558–66. doi:10.1002/lary.29206

34. McLigeyo A, Owuor K, Ng'ang'a E, Mwangi J, Wekesa P. Characteristics and Treatment Response of Patients with HIV Associated Kaposi's Sarcoma in Central Kenya. HIVAIDS - Res Palliat Care. 2022 Apr;Volume 14:207–15. doi:10.2147/HIV.S359278

35. Mduma E, Dharsee N, Samwel K, Mwita CJ, Lidenge SJ. Clinicopathological Characteristics and Outcomes of Anal Squamous Cell Carcinoma Patients With and Without HIV Infection in Sub-Saharan Africa. JCO Glob Oncol. 2023;9:e2200394. doi:10.1200/GO.22.00394

36. Moahi K, Ralefala T, Nkele I, Triedman S, Sohani A, Musimar Z, et al. HIV and Hodgkin Lymphoma Survival: A Prospective Study in Botswana. JCO Glob Oncol. 2022;8:e2100163. doi:10.1200/GO.21.00163

37. Mrema AS, Ngoma M, Josiah C, Lugina E, Mvungi N, Paul M, et al. HIV and Early Treatment Outcomes Among Women With Cervical Cancer Treated With Concurrent Chemoradiation in Tanzania. JCO Glob Oncol. 2023;9:e2200441. doi:10.1200/GO.22.00441

38. Musekwa E, Chapanduka ZC, Bassa F, Kruger M. An 8-year retrospective study of adult and paediatric Burkitt's lymphoma at Tygerberg Hospital, South Africa. SA J Oncol. 2020. doi:10.4102/sajo.v4i0.93

39. Okello CD, Omoding A, Ddungu H, Mulumba Y, Orem J. Outcomes of treatment with CHOP and EPOCH in patients with HIV associated NHL in a low resource setting. BMC Cancer. 2020;20(1):798. doi:10.1186/s12885-020-07305-2

40. Phakathi BP, Basson G, Karusseit VOL, Olorunju SAS, Mokoena T. The effect of HIV infection on the surgical, chemo- and radiotherapy management of breast cancer. A prospective cohort study. Int J Surg. 2016. doi:10.1016/j.ijsu.2016.08.520

41. Phipps W, Adams SV, Mooka P, Kafeero J, Sekitene S, Mubiru D, et al. A prospective study of clinical outcomes of HIV-associated and HIV-negative Kaposi sarcoma in Uganda. AIDS. 2023;37(1):51–9. doi:10.1097/QAD.0000000000003376

42. Rapiti N, Peer N, Abdelatif N, Rapiti P, Moosa Y. HIV-associated plasmablastic lymphoma: A single-centre 12-year experience in Kwa-Zulu Natal, South Africa. HIV Med. 2022;23(8):837–48. doi:10.1111/hiv.13266

43. Sengayi M, Kielkowski D, Egger M, Dreosti L, Bohlius J. Survival of patients with Kaposi sarcoma in the South African antiretroviral treatment era: a retrospective cohort study. S Afr Med J. 2017;107(10):871–6. doi:10.7196/SAMJ.2017.v107i10.12362

44. Turdo YQ, Ruffieux Y, Boshomane TMG, Mouton H, Taghavi K, Haas AD, et al. Cancer treatment and survival among cervical cancer patients living with or without HIV in South Africa. Gynecol Oncol Rep. 2022;43:101069. doi:10.1016/j.gore.2022.101069

45. Vogt SL, Laudin G, Zahurak M, Vaughan J, Lakha A, Pather S, et al. Real-world treatment outcomes for Hodgkin lymphoma in South Africa: a prospective observational study. Infect Agent Cancer. 2024;19(1):46. doi:10.1186/s13027-024-00612-4

46. Bhatia RK, Rayne S, Rate W, Bakwenabatsile L, Monare B, Anakwenze C, et al. Patient Factors Associated With Delays in Obtaining Cancer Care in Botswana. J Glob Oncol. 2018;4:1–13. doi:10.1200/JGO.18.00088

47. Montaño MA, Jatho A, Nassolo C, Mugisha N, Bula A, Chagomerana MB, et al. Healthcare provider perspectives on integrating HIV care into cancer centers in Malawi, South Africa, Uganda, and Zimbabwe. Transl Oncol. 2025;53:102273. doi:10.1016/j.tranon.2025.102273

48. Idemili-Aronu N, Okoli IA, Jemisenia JO, Olawepo JO, Onyeka T, Ezeanolue EE. Healthcare providers' awareness, perceptions and experiences in integrating palliative care for women with HIV and cancer in Nigeria: a qualitative study on barriers and facilitators. BMC Health Serv Res. 2026;26(1):223. doi:10.1186/s12913-026-14029-9

49. Martei YM, Obasohan M, Mokokwe L, Ralefala T, Mosepele M, Gross R, et al. Stigma and Social Determinants of Health Associated With Fidelity to Guideline-Concordant Therapy in Patients With Breast Cancer Living With and Without HIV in Botswana. The Oncologist. 2023;28(12):e1230–8. doi:10.1093/oncolo/oyad183

50. McMahon DE, Singh R, Chemtai L, Semeere A, Byakwaga H, Grant M, et al. Barriers and facilitators to chemotherapy initiation and adherence for patients with HIV-associated Kaposi's sarcoma in Kenya: a qualitative study. Infect Agent Cancer. 2022;17(1):37. doi:10.1186/s13027-022-00444-0

51. Nyagabona SK, Haddadi S, Mtei RJ, Sunguya B, Kahesa C, Pallangyo CK, et al. Paving the Way for an Integrated Care Model: Experiences of Women Living With HIV and Breast Cancer in Tanzania. JCO Glob Oncol. 2025;11:e2500330. doi:10.1200/GO-25-00330
